# Design of 20-deoxyingenol-esters-based PKC agonists and their lysosome biogenesis-enhancing activity

**DOI:** 10.1007/s13659-025-00522-x

**Published:** 2025-06-10

**Authors:** Jia-Jia Wan, Qiu-Yuan Yin, Mao Sun, Cui-Shan Zhang, Hao-Jing Zang, Pei-Tong Yao, Ming-Rui Yuan, Ding-Kang Chen, Feng Guo, Qun Chen, Bo-Wen Ouyang, Zi-Fei Xu, Ming-Ming Cao, Chong-Lin Yang, Xiao-Jiang Hao, Ying-Tong Di

**Affiliations:** 1 State Key Laboratory of Phytochemistry and Plant Resources in West China, Kunming Institute of Botany, Chinese Academy of Sciences, Kunming, 650201, China; 2 Yunnan Key Laboratory of Natural Medicinal Chemistry, Kunming, 650201 China; 3 University of Chinese Academy of Sciences, Beijing, 100049 China; 4 School of Life Sciences, Yunnan University, Kunming, 650091 China; 5 An Shun City People’s Hospital, Anshun, 561000 China; 6 Research Unit of Chemical Biology of Natural Anti-Virus Products, Chinese Academy of Medical Sciences, Beijing, 100730 China

**Keywords:** 20-deoxyingenol, SAR, PKCδ, Amino acid

## Abstract

The activation of conventional (α) and novel (δ) protein kinase C (PKC) isoforms promotes lysosomal biogenesis, a critical process for clearance of pathogenic protein aggregates including β-amyloid (Aβ) and phosphorylated Tau (p-Tau) in neurodegenerative disorders. Notably, PKC activators HEP14/15, characterized by 20-methyl moiety, fail to establish classical C1B domain pharmacophore interactions, suggesting a non-canonical activation mechanism. In this study, structural diversification of 20-deoxyingenol through esterification and acetonide protection yielded 18 new derivatives (**2–19**). Systematic screening revealed their lysosome-promoting activities, with structure–activity relationship analysis identifying compounds **4** and **18** as superior autophagy inducers. At 20 μM, these derivatives enhanced autophagic flux by 2.45-fold and 2.31-fold versus vehicle control. Moreover, compounds **4** and **18** exhibited a dose-dependent increase in lysosome numbers, promoted TFEB nuclear translocation, and enhanced lysosome-mediated lipid droplet clearance. Western blot analysis further revealed that compounds **4/18** upregulated proteins associated with the autophagy-lysosome system, suggesting their potential as promising autophagy inducers. Mechanistically, molecular docking simulations indicated thier high-affinity binding to PKCδ, which may explain their autophagy-enhancing properties.

**Graphical Abstract:**

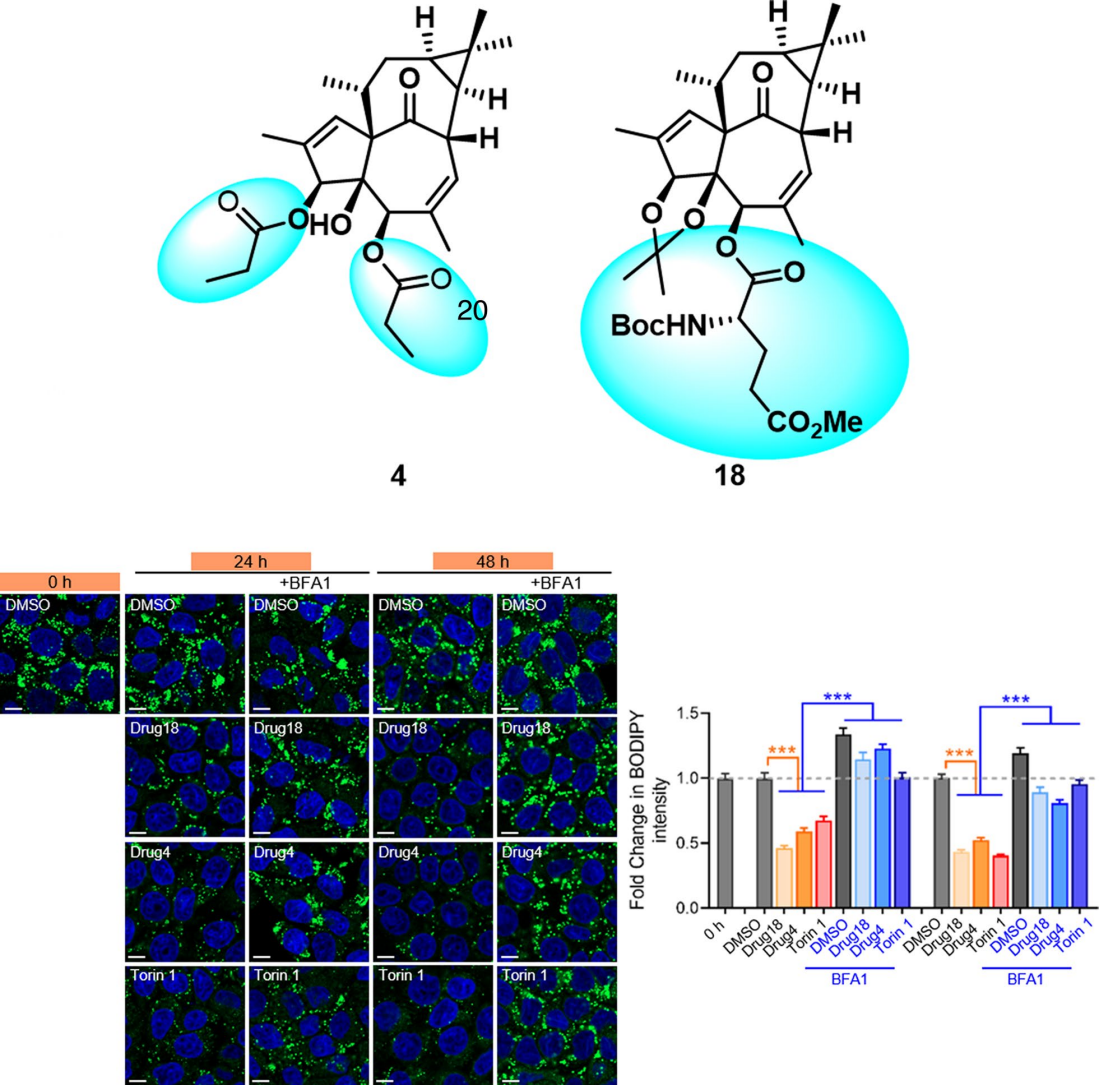

**Supplementary Information:**

The online version contains supplementary material available at 10.1007/s13659-025-00522-x.

## Introduction

The Protein Kinase C (PKC) family, as core members of the serine/threonine kinase superfamily, has been widely validated for its pivotal role in regulating cell fate decisions (proliferation, differentiation, apoptosis) and metabolic homeostasis [1]. Based on structural features and activation mechanisms, PKC isoforms can be classified into three categories: conventional PKCs (cPKCs: α, βI/II, γ), novel PKCs (nPKCs: δ, ε, η, θ), and atypical PKCs (aPKCs: ζ, ι/λ) [2, 3]. The classical and novel PKCs (c/nPKCs) are particularly noteworthy, whose N-terminal regulatory domains contain C1A/C1B modules that function as molecular biosensors for diacylglycerol (DAG) [4–6]. This critical step has become a focal point for drug design targeting cancer, neurodegenerative diseases, and other conditions. Recent crystal structure analyses (e.g., the C1δ-DAG complex) have further precisely identified ligand-binding sites, providing an atomic-level blueprint for structure-based rational drug development [7].

**Scheme 1:**
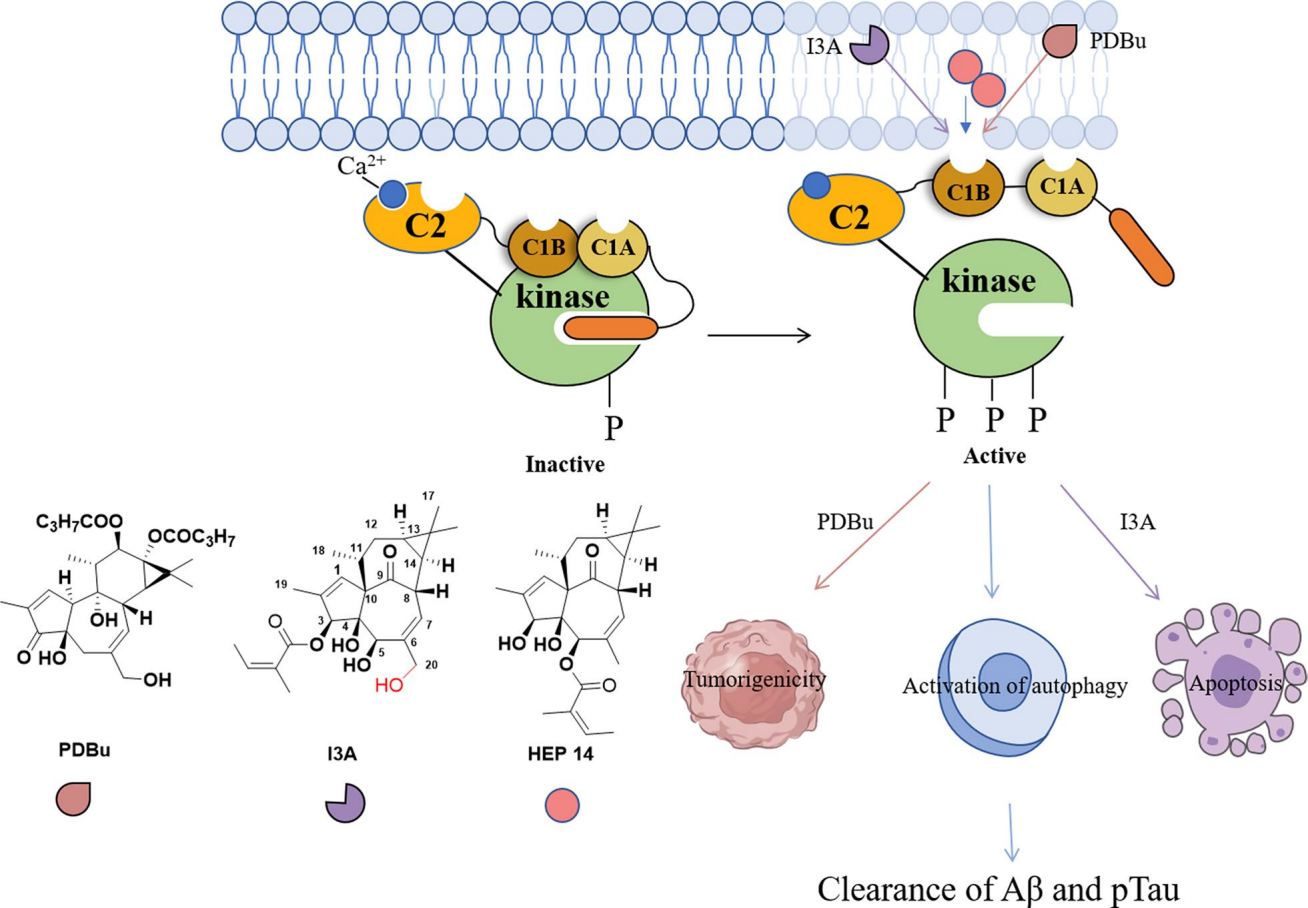
Differential engagement of DAG-binding sites by PKC agonists elicits distinct cellular responses

Natural products serve as rich sources of protein kinase C (PKC) modulators, with *Euphorbia* diterpenoids exhibiting unique structural architectures and diverse bioactivities [8–18]. Phorbol esters [8, 9] and ingenol 3-angelate (I3A) [14], for instance, activate PKC signaling by mimicking diacylglycerol (DAG) binding to the PKC C1 domain, while anchoring their 20-hydroxymethyl groups to form stabilizing hydrogen-bond networks. Intriguingly, these structural homologs exhibit divergent biological effects: I3A demonstrates antitumor properties, whereas phorbol 12,13-dibutyrate paradoxically promote carcinogenesis (Scheme 1) [19, 20]. All the data implied that molecular variations can drive diversified therapeutic outcomes in PKC agonists.

Emerging as a novel PKC modulator, 20-deoxyingenol ester exhibits unique isoform selectivity and significant therapeutic potential. Unlike many kinase-targeting compounds, it bypasses the mechanistic target of rapamycin (mTOR)—a serine/threonine kinase that assembles into distinct mTORC1 and mTORC2 complexes with divergent structures and functions—and instead selectively activates the PKCα and PKCδ isoforms [21]. This selective activation triggers two parallel signaling cascades: activation of the TFEB transcription factor and inactivation of the ZKSCAN3 transcriptional repressor, which collectively drive lysosome biogenesis [13]. Importantly, 20-deoxyingenol ester demonstrates minimal cytotoxicity, no observed tumorigenicity, and robust β-amyloid (Aβ) clearance efficacy in both cellular models and Alzheimer’s disease (AD) murine systems [13]. These properties highlight its translational potential, making further elucidation of its structure–activity relationship (SAR) a high-priority research objective.

However, natural 20-deoxyingenol derivatives are constrained by limited availability and structural diversity [22]. To overcome these bottlenecks, our study employs 20-deoxyingenol as a molecular scaffold for systematic chemical modifications at the C3, C4, and C5 positions. Using lysosome biogenesis activity as a functional readout, we aim to establish PKC-binding SAR profiles while integrating molecular docking to dissect ligand-C1 domain interactions. This integrative approach not only accelerates lead compound discovery for neurodegenerative diseases but also redefines the framework for C1-targeted drug discovery.

## Results

To investigate the effects of substituents at positions 3/4/5 on biological activity, we developed two structural modifications to enhance potency: (A) Diacylated derivatives and (B) Monoacylated derivatives followed by acetonide protection of vicinal diols. This study evaluates explicitly the impact of these modified compounds on autophagy-activating activity.

**Scheme 2:**
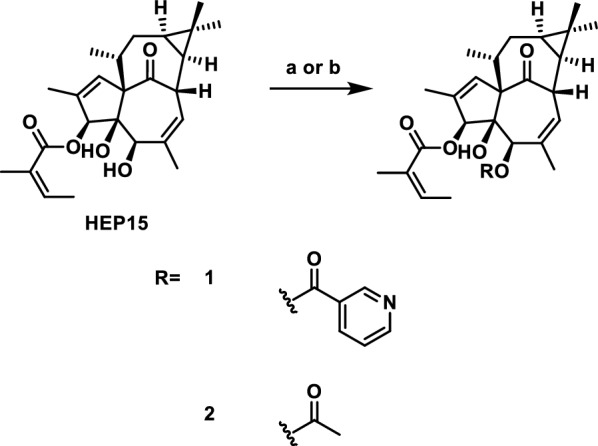
Reagents and conditions: **a** ROH (10 eq*.*), DMAP (10 eq*.*), EDCI (10 eq*.*), DCM, r.t. **b** RCl (10 eq*.*), DMAP (10 eq*.*), Et_3_N, DCM, 40 ℃

**Fig. 1:**
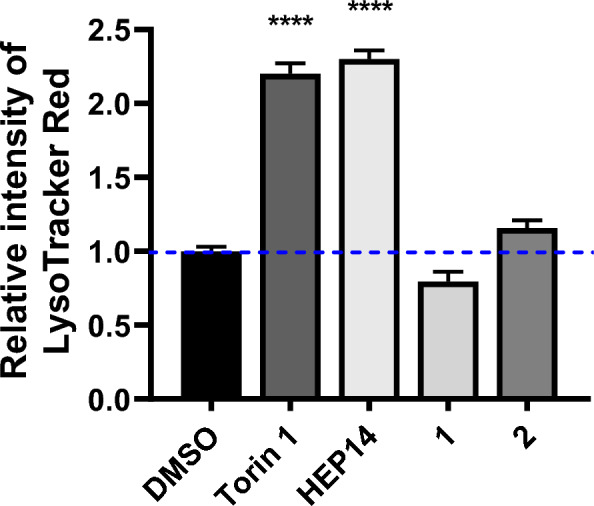
Inducing autophagy-flow activity of ester derivatives. The tested concentration was 20 μM. Torin 1 (2 μM) as positive control. Data are representative of three independent experiments. *P < 0.05, ****P < 0.0001 vs the DMSO

We first used **HEP15** as a substrate for acylation (Scheme 2), selecting nicotinoyl and acetyl groups to test the effects of products **1**/**2** on lysosome biogenesis. We constructed human HeLa cells to estimate autophagy activity systemically. As shown in Fig. 1, compound **2** promotes lysosome biogenesis, indicating that the C1 domain of PKC can accommodate smaller diacylated products, which was also supported by docking experiments. In contrast, compound **1** inhibited this activity. Although the exact mechanism remains unclear, the bulkier nicotinoyl group in **1** likely prevents its entry into the C1B domain of PKCδ, thereby interfering with PKC membrane-binding process and disrupting normal lysosomal turnover.

**Scheme 3:**
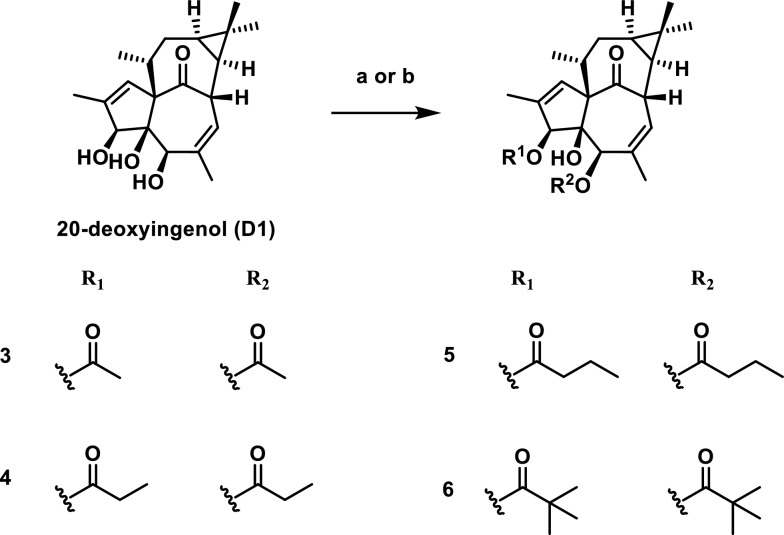
Reagents and conditions: **a** ROH (10 eq.), DMAP (10 eq*.*), EDCI (10 eq*.*), DCM, r.t. **b** RCl (10 eq*.*), DMAP (10 eq*.*), Et_3_N, DCM, 40 ℃

**Scheme 4:**
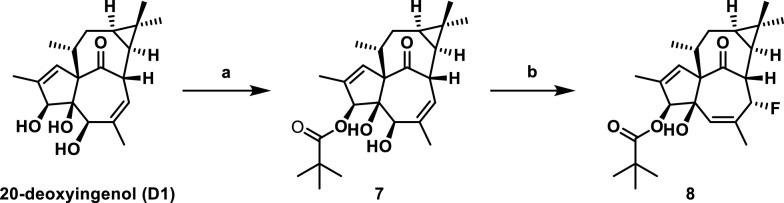
Synthesis of **7**, **8**. (a) PivCl (10 eq*.*), DMAP (10 eq*.*), Et_3_N, DCM, 40 ℃. (b) DSAT, DCM, 0℃ to r.t

Subsequently, to further investigate the effect of acyl chain length on lysosome biogenesis, we acylated substrate 20-deoxyingenol (D1) using acetyl, propionyl, butyryl, pivaloyl, nicotinoyl, and Boc-protected isoleucinyl as acylating agents (Scheme 3). Since both the 3- and 5-positions of D1 can undergo acylation, all mono-acylated products except the pivaloylated derivative underwent migration during isolation and storage, making it impossible to obtain pure mono-acylated products. Therefore, we synthesized 3,5-diacylated 20-deoxyingenol esters. Additionally, we successfully isolated the 3-mono-pivaloylated 20-deoxyingenol esters **7** and **8** (Scheme 4).

**Fig. 2:**
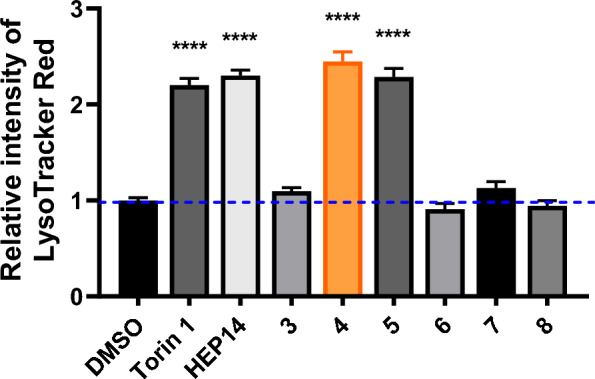
Inducing autophagy-flow activity of ester derivatives. The tested concentration was 20 μM. Torin 1 (2 μM) as positive control. Data are representative of three independent experiments. *P < 0.05, ****P < 0.0001 vs the DMSO

As shown in Fig. 2, activity screening revealed that among these diacylated products, the disubstituted propionylated derivative **4** exhibited the highest activity, surpassing even the positive control **HEP14**. Additionally, compound **5** has a slightly longer side chain than compound **4**, yet exhibits marginally lower activity. While compound **7** demonstrates detectable activity, its potency shows no significant difference compared to the control group and remains substantially lower than that of compound **4**. We further derivatized this compound to generate **8** by removing the 5-hydroxyl group and introducing a fluorine atom at position **7** to enhance lipophilicity. However, this structural modification resulted in complete loss of autophagy-activating activity.

**Scheme 5:**
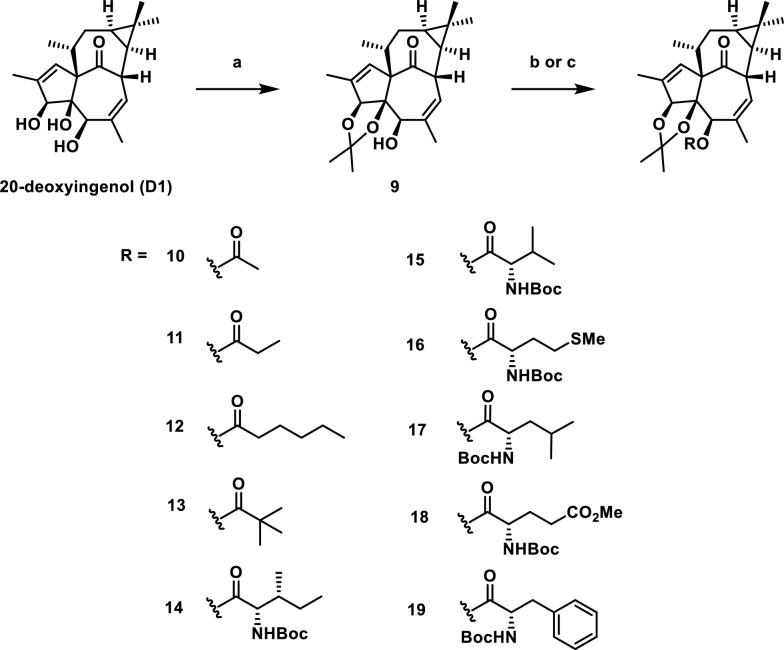
Synthesis of 5-*O*-acyl-20-ingenol (**9**–**19**). Reagents and conditions: **a** 2,2-dimethoxypropane (4.8 eq.), PTSA (0.05 eq*.*), acetone, r.t; **b** ROH or Boc-L-Ile (10 eq*.*), DMAP (10 eq*.*), EDCI (10 eq*.*), DCM, r.t. **c** RCl (10 eq*.*), DMAP (10 eq*.*), Et_3_N, DCM, 40 ℃

**Fig. 3:**
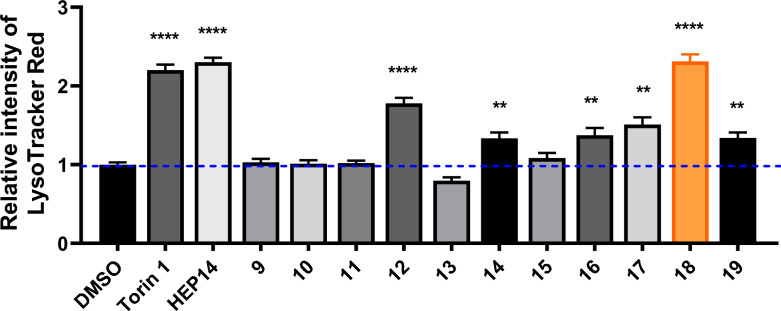
Inducing autophagy-flow activity of ester derivatives. The tested concentration was 20 μM. Torin 1 (2 μM) as positive control. Data are representative of three independent experiments. *P < 0.05, ****P < 0.0001 vs the DMSO

If diacylated products are active, can monoacylated derivatives also activate autophagy? Given the propensity for acyl group migration between the 3- and 5-positions, we first protected the adjacent hydroxyl groups with an acetonide moiety. This exclusively yielded the 3,4-acetonide-protected product, with no observed formation of the 4,5-protected analogue. Based on this scaffold, we synthesized a series of acylated derivatives with varying chain lengths (compounds **9–13**, Scheme 5). Biological evaluation revealed that compound **12**, bearing an acyl group with more than three carbons, retained autophagy activation capability; however, its activity was markedly lower than that of the positive controls Torin 1 and **HEP 14** (Fig. 3). Notably, this observation aligns with the previously reported trend in saturated linear esters, where increased ester chain length correlated with improved HIV-1 latency reversal activity [5]. Comparative analysis of inactive compounds (**9–11** and **13**) suggests that their shared acetonide group is unlikely to act as the catalytically active moiety.

Subsequently, we investigated the effects of different amino acid residues on activation (compounds **14**–**19**). As shown in Fig. 3, the majority of compounds displayed potency of inducing autophagy activity, whereas compound **15** showed almost no biological activity. In contrast, compound **18**, the protected derivative of methylglutamic acid, was the best potent compound to promote autophagy-inducing activity at a 2.31-fold than control. Notably, its activity surpassed that of the positive control Torin 1 at 2 μM (2.20-fold) and reached levels comparable to **HEP14**. In addition, compounds **14**, **16**, **17**, and **19** exhibited weaker activity compared to **18**. This suggested that introduction of amino acid with larger size groups such as methyl groups and aromatic ring on the terminal of side chain could decreased the activity.

**Fig. 4:**
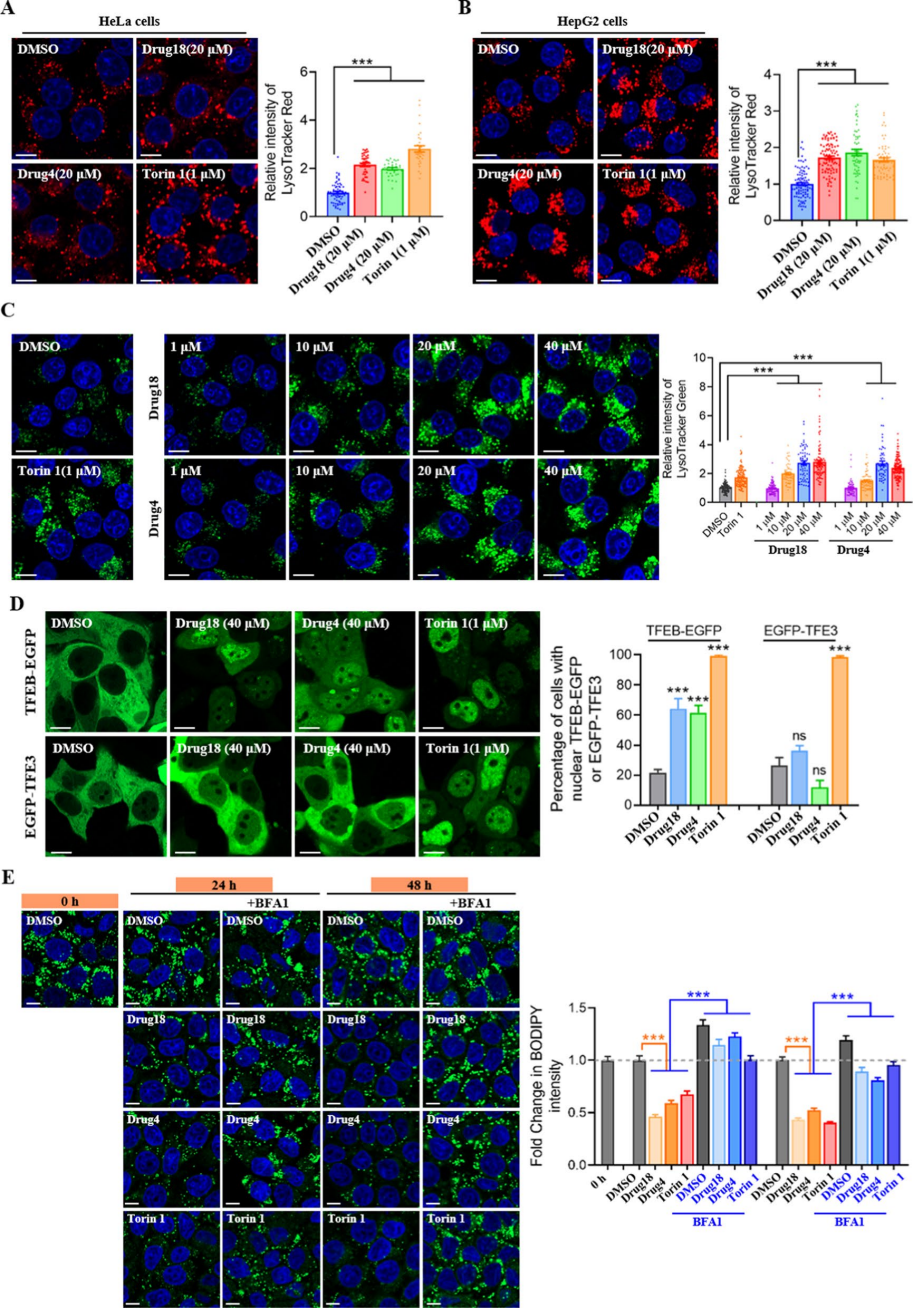
(A and B) Images (left) and quantifications (right) of endogenous Lyso Tracker Red in HeLa (**A**) and HepG2 (**B**) cells treated for 3 h with Drugs (20 μM) or Torin1 (1 μM). n = 3 independent experiments. Bars represent 10 μm in all images except. **C** Images (left) and quantifications (right) of endogenous Lyso Tracker Green in HeLa cells treated for 3 h with Drugs (1–40 μM). n = 3 independent experiments. **D** Images (left) and quantification (right) of the subcellular locations TFEB-EGFP or EGFP-TFE3 in HeLa cells treated with Drugs (40 μM, 3 h). n = 3 independent experiments. **E** Images (left) and quantifications (right) of HeLa cells treated with Drugs (20 μM, 3 h) and co-stained with BODIPY-pepstatin A (1 μM). n = 3 independent experiments. Data (mean ± s.e.m) were compared using t-tests or ANOVA ^∗∗^*p* < 0.01, ^∗∗∗^*p* < 0.001

Our previous study demonstrated that **HEP14/15** induces TFEB-dependent lysosome biogenesis. To determine whether **4** and **18** exert similar effects, we treated HeLa and HepG2 cells with these compounds for 3 h. LysoTracker Red staining revealed a significant increase in lysosome numbers following treatment with **4** and **18** (Fig. 4A, B), and this effect was dose-dependent (Fig. 4C). Furthermore, both compounds **4** and **18** promoted the nuclear translocation of TFEB in cells ectopically expressing TFEB-EGFP, whereas the subcellular localization of TFE3 remained largely unchanged (Fig. 4D).

Given that **4** and **18** induce lysosome biogenesis, we next investigated whether these compounds enhance lysosome-dependent cellular clearance. In HepG2 cells overloaded with oleic acid to induce lipid droplet formation, treatment with **4** and **18** led to a reduction in lipid droplet numbers. However, in the presence of bafilomycin A1 (BFA1), an inhibitor of lysosomal degradation, neither **4** nor **18** reduced lipid droplet numbers. These findings indicate that **4** and **18** promote lysosome-dependent clearance of lipid droplets (Fig. 4E).

**Fig. 5:**
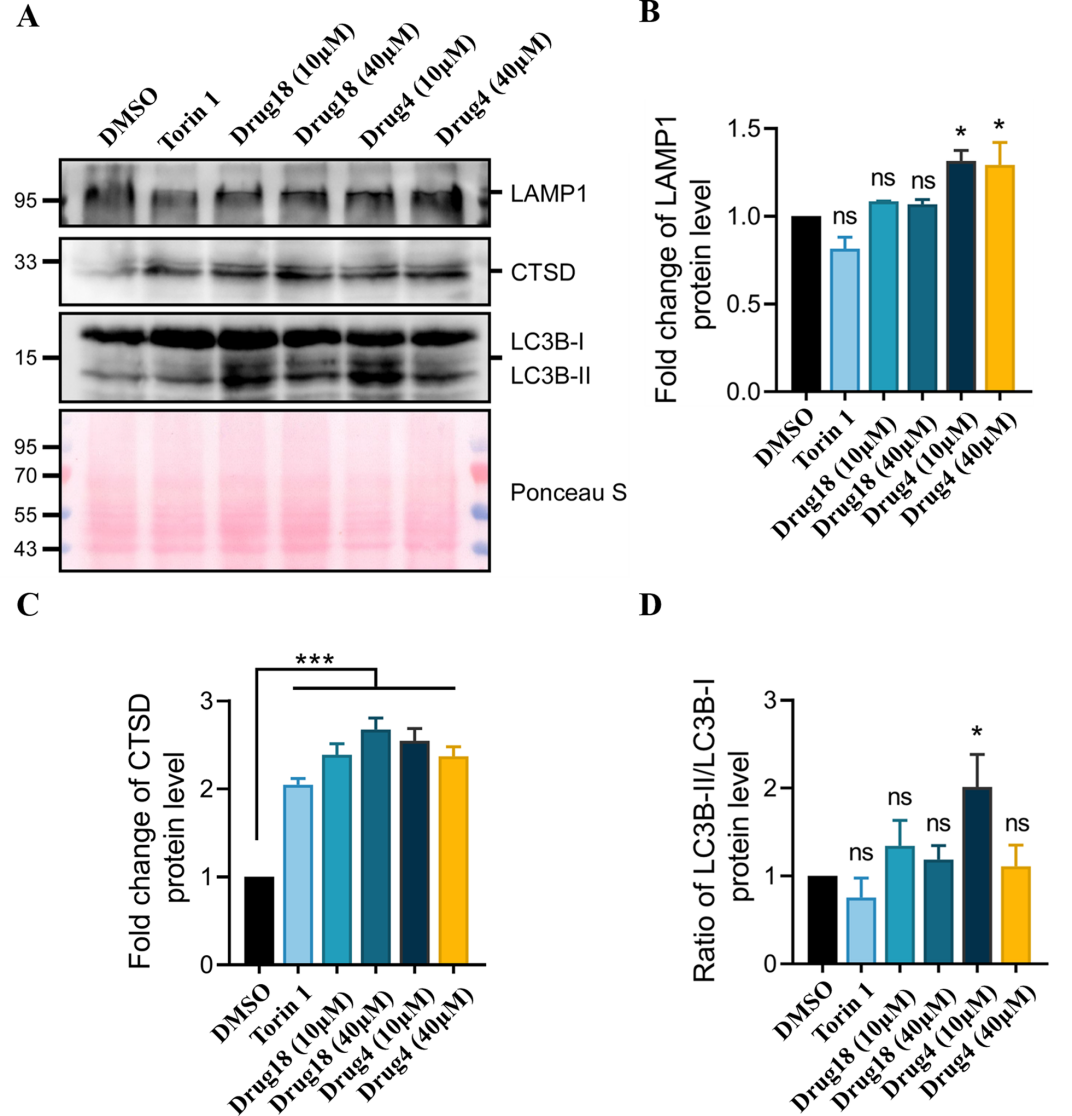
The autophagy-lysosome system was activated by **4**. **A** Representative Western blot result showed the protein levels of LAMP1, CTSD, LC3-II/LC3-I and Ponceau S staining in the HeLa cells treated with **4, 18** or Torin 1. **B**–**F** Quantifications of the protein levels in the HeLa cells in a based on two independent experiments. The one-way ANOVA with the post hoc Holm-Sidak test was used to detect the expression differences between groups, and the values were expressed as the mean ± SEM. *, *P* < 0.05; **, *P* < 0.01; ***, *P* < 0.001; ****; ns, not significant

To further verify that **4** and **18** enhances autophagy, we also assessed the expression of proteins related to autophagy-lysosome system by Western blot. The human cervical cancer cell line (HeLa cells) was treated with the compounds **4** and **18** for 24 h. Then, the proteins were extracted after cell lysis. We used Dimethyl Sulfoxide (DMSO) as a control and Torin 1, an autophagy inducer, as a positive control. The protein level of lysosomal-associated membrane protein 1 (LAMP1) was increased in a dose-dependent manner in response to **4** (Fig. 5A, B). Moreover, the protein level of cathepsin D (CTSD) which are important protease in lysosomes were also increased (Fig. 5A, C). These results indicate that lysosome function is enhanced. In addition, **4** increased the protein level of the lipidated (PE-conjugated) form of MAP1LC3/LC3 (microtubule-associated protein 1 light chain 3 beta; LC3-II):LC3-I (Fig. 5A, D), which partially indicated that autophagy was enhanced. Collectively, these results suggest that compound **4** can activate autophagy-lysosome system and might be a promising autophagy inducer.

**Fig. 6:**
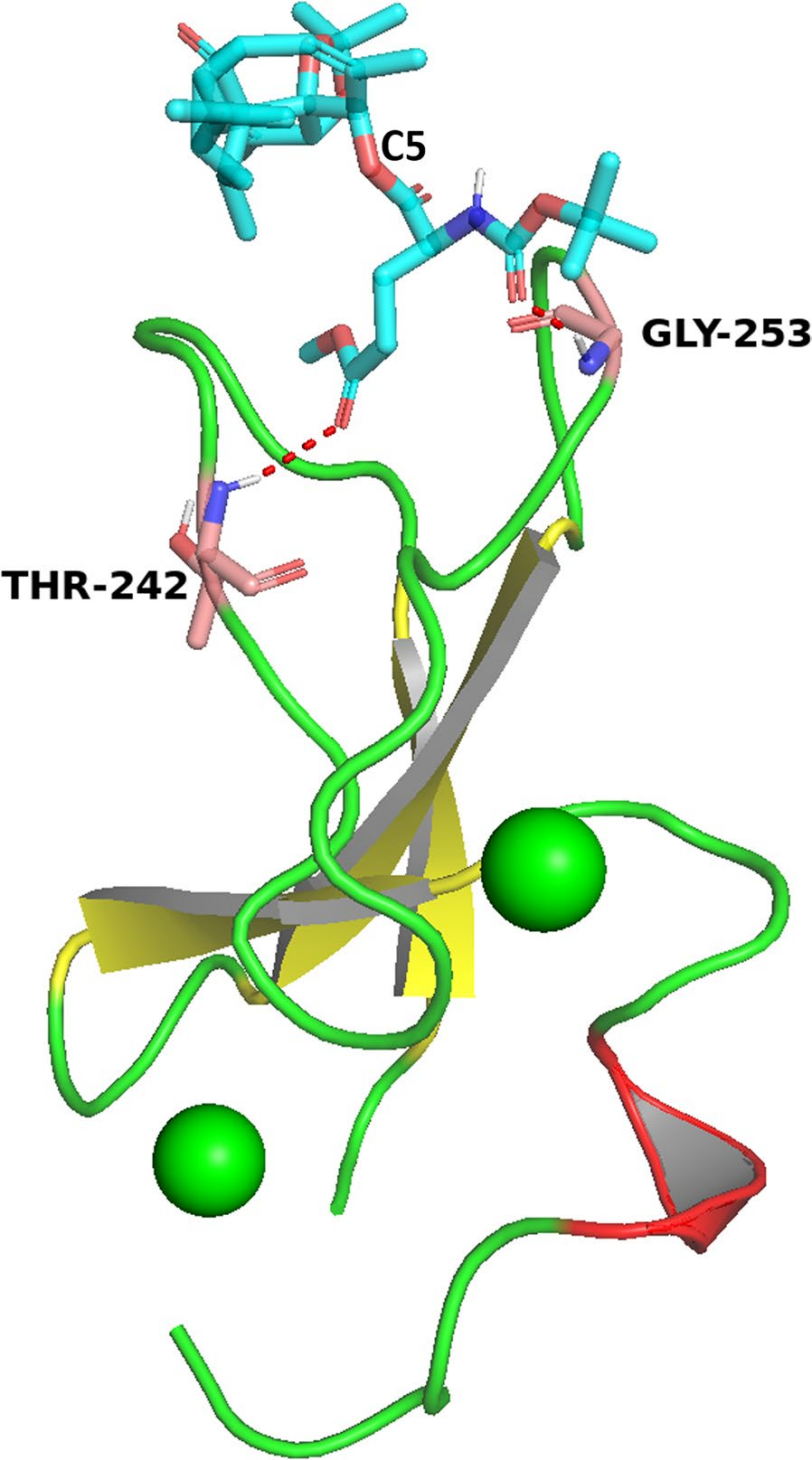
Predicted binding mode of compound **18** with PKCδ C1B domain (PDB code: 1PTR). The oxygen atoms and nitrogen atoms are shown in red and blue, respectively. The yellow dotted line indicates possible hydrogen bond. The figures were generated using PyMol (https://www.schrodinger.com/products/pymol)

Previous study reported that **D1** ester promoted autophagy by binding to PKCδ C1B domain [13]. Thus, molecular docking was employed to further analyze the interaction of **18** with PKC (Fig. 6). According to binding result, the methyl eater of L-Glu was anchored to biding site of the activity pocket and protective group located on the surface of protein. Two hydrogen bonds were formed between **18** and δC1B domain: a hydrogen bond between the C = O group of methyl ester and the NH group of THR 242 at 2.50 Å, another between the C = O group of the protective group and NH group of Gly253 at 1.95 Å. The *t*-butyl ester and methyl groups on the rigid ring skeleton displayed hydrophobic interaction with the hydrophobic residues in present in the upper portion [23]. In addition, it is required for PKC activation that ligand’s hydrophobic segment inserted into cell membrane [24, 25]. The simulation model suggested that the rigid rings exposure outside of protein might be inserted in the phospholipid bilayers and help PKC anchoring to cell membrane [23, 24]. Therefore, supported by docking study and bioassay results, we concluded that **18** might interact with PKC to possessed autophagy-inducing activity might be based on binding with PKCδ.

## Discussion

Protein kinase C (PKC) represents a compelling therapeutic target for Alzheimer's disease (AD) due to its ability to regulate multiple neuroprotective pathways. While conventional PKC activators such as diacylglycerol (DAG) induce transient activation, natural product-derived PKC agonists often exhibit prolonged bioavailability, leading to sustained PKC activation, aberrant apoptotic signaling, and oncogenic risks—effects absent in physiological DAG-dependent signaling. This highlights the need for PKC activators with weaker binding affinity and fast dissociation kinetics to mimic endogenous DAG's transient activation profile while avoiding pathological overactivation [26–29].

Our findings redefine the pharmacophore requirements for PKC activation, emphasizing: 1). Kinetic control: Fast-dissociating agonists avoid sustained PKC overactivation; 2). Membrane recruitment: Hydrophobic interactions drive PKC localization and signaling. This paradigm shift opens new avenues for developing safer PKC-targeted therapeutics for AD and other neurodegenerative diseases, where balanced pathway activation is critical.

Unlike traditional diterpenoid PKC agonists, these compounds avoid prolonged kinase activation while retaining therapeutic efficacy. This kinetic profile positions them as promising candidates for further derivatization to optimize PKC engagement without the risks associated with conventional PKC activators.

## Experimental

### General experimental procedure

Unless otherwise mentioned, all reagents and catalysts were purchased from commercial suppliers and used without purification. Analytical TLC systems were carried out on silica gel 60 F254 plates Silica gel. Column chromatography (CC) was performed using silica gel (200–300 mesh and 60–80 mesh). The NMR spectra were obtained in CDCl_3_ at ambient temperature on were on Bruker DRX-500 and DRX-600 instruments with DMSO-*d6* as solvent unless otherwise indicated.. High-resolution MS data were performed on an Agilent 1290 UPLC/6540 Q-TOF mass spectrometer in positive mode. Spectra were referenced internally to the residual proton resonance in CDCl_3_(7.26 ppm for ^1^H and 77.0 ppm for ^13^C) as the internal standard. Coupling constants (*J*) were reported in hertz (Hz).

### The preparation of 3,5-*O*-diester-20-deoxyingenol (1–5)

To the solution of **20-deoxyingenol** (0.134 mmol) in dry 1,2-Dichloroethane (10 mL), DMAP (1.34 mmol), corresponding acid (1.34 mmol) and EDCI (1.34 mmol) were added. The mixture was stirred at room temperature for 4 h. The reaction was monitored by TLC, and the solvent was removed under reduced pressure. The residue was purified by column chromatography (EtOAc/PE) to give the compounds in good yield.

### The preparation of 6–7

To the solution of **20-deoxyingenol** (87 mg, 0.262 mmol) in dry 1,2-Dichloroethane (10 mL), DMAP (64 mg, 0.524 mmol), Et_3_N (728 μL, 5.24 mmol), Pivaloyl chloride (322 μl, 2.620 mmol) were added. The mixture was heated to reflux at 40 ℃ for 16 h. The solvent was removed under reduced pressure. The residue was solved in EtOAc (40 mL) and washed with water (20 mL × 3) and saturated NaCl (20 mL × 2). The combined organic faction were concentrated under reduced pressure and the residue purified by column chromatography (EtOAc:PE = 12:1) to give the **6** (101.7 mg, yield 74%); purified by column chromatography (EtOAc:PE = 4:1) to give the **7** (28.9 mg, yield 25%).

### The preparation of fluoride (8)

20-mL teflon vial fitted with a rubber septum was charged with **13** (7 mg, 0.017 mmol). The reaction vessel was evacuated and refilled using a balloon of argon. This process was repeated three times. Dry 1,2-dichloroethane (2 mL) were added to the reaction vessel via syringe and the resulting mixture was cooled to 0 °C. Deoxo-Fluor® (6.3 μL, 0.034 mmol) was added. The reaction mixture was stirred at 0 °C for 12 h and the ice bath was removed. The mixture was quenched with saturated NaHCO_3_ and transferred to a separatory funnel that had been charged with ethyl acetate (10 mL). Then, the resulting mixture was washed with water (5 mL × 3) and saturated NaCl (20 mL × 2). The organic layers were dried with dry MgSO_4_ and purified by column chromatography (EtOAc:PE = 30:1) to give the **15** as a white solid (3.1 mg, 44%).

### The preparation of intermediate (20-deoxyingenol-3,4-acetonide, 9)

To the solution of **20-deoxyingenol** (100 mg, 0.30 mmol) in acetone (10 ml) was added 2,2-Dimethoxypropane (591 μL, 1.44 mmol), p-Toluenesulfonicacid (31 mg, 0.18 mmol) were added. The mixture was stirred in room temperature for 5 h. NaHCO3 (16 mg, 0.18 mmol) was added to keep the pH = 7. Then the mixture was extracted with ethyl acetate (3 × 50 mL), washed with saturated NaCl (2 × 50 mL). The combined organic extract was dried over anhydrous NaSO4, filtered and the solvent was removed under reduced pressure. Column chromatography of the product on silica gel (EtOAc:PE = 15:1) gave a white solid (104 mg, 93%).

### General method for the preparation of compounds 10–19

As described in Scheme 4, to the solution of **20-deoxyingenol** (0.134 mmol) in dry 1,2-Dichloroethane (10 mL), DMAP (1.34 mmol), corresponding acid (1.34 mmol) and EDCI (1.34 mmol) were added. The mixture was stirred at room temperature for 3 h. The reaction was monitored by TLC, and the solvent was removed under reduced pressure. The residue was purified by column chromatography (EtOAc/PE) to give the compounds in good yield.

## Supplementary Information

Additional file 1.

Additional file 2.

## Data Availability

Data will be made available on reasonable request.
