# Correction: Design of 20‑deoxyingenol‑esters‑based PKC agonists and their lysosome biogenesis‑enhancing activity

**DOI:** 10.1007/s13659-025-00537-4

**Published:** 2025-08-19

**Authors:** Jia‑Jia Wan, Qiu‑Yuan Yin, Mao Sun, Cui‑Shan Zhang, Hao‑Jing Zang, Pei‑Tong Yao, Ming‑Rui Yuan, Ding‑Kang Chen, Feng Guo, Qun Chen, Bo‑Wen Ouyang, Zi‑Fei Xu, Ming‑Ming Cao, Chong‑Lin Yang, Xiao‑Jiang Hao, Ying‑Tong Di

**Affiliations:** 1 State Key Laboratory of Phytochemistry and Plant Resources in West China, Kunming Institute of Botany, Chinese Academy of Sciences, Kunming, 650201 China; 2 Yunnan Key Laboratory of Natural Medicinal Chemistry, Kunming, 650201 China; 3 University of Chinese Academy of Sciences, Beijing, 100049 China; 4 School of Life Sciences, Yunnan University, Kunming, 650091 China; 5 An Shun City People’s Hospital, Anshun, 561000 China; 6 Research Unit of Chemical Biology of Natural Anti‑Virus Products, Chinese Academy of Medical Sciences, Beijing, 100730 China

**Correction: Natural Products and Bioprospecting (2025) 15:38** 10.1007/s13659-025-00522-x

Following publication of the original article [1], the co-first authorship has been updated: Jia Jia Wan, Qiu Yuan Yin, Mao Sun, and Cui Shan Zhang contributed equally to this work and share co-first authorship.

The original article [1] has been updated.
