# Supplementary material for: Design of 20-deoxyingenol-esters-based PKC agonists and their lysosome biogenesis-enhancing activity
Source: Nat Prod Bioprospect. 2025 Jun 10;15(1):38. doi: 10.1007/s13659-025-00522-x (PMC12151967; doi:10.1007/s13659-025-00522-x)
Supplement: Supplementary file 1 — Additional file 1. [file 13659_2025_522_MOESM1_ESM.pdf]

Supporting Information

Design of 20-Deoxyingenol-esters-Based PKC Agonists and Their Lysosome Biogenesis-enhancing Activity

Jia-Jia Wan[a,b,1], Qiu-Yuan Yin[c,1], Mao Sun[d,1], Cui-Shan Zhang[a,b,1], Hao-Jing Zang[a,c], Pei-Tong Yao[a,b], Ming-Rui Yuan[a,b], Ding-Kang Chen[a,b], Feng Guo[a,b], Qun Chen [a,c], Bo-Wen Ouyang[a,b], Zi-Fei Xu[a], Ming-Ming Cao[a], Chong-Lin Yang[c]*, Xiao-Jiang Hao[a,b,e]* and Ying-Tong Di [a,b]*

[a] *State Key Laboratory of Phytochemistry and Plant Resources in West China, Kunming Institute of Botany, Chinese Academy of Sciences; Yunnan Key Laboratory of Natural Medicinal Chemistry, Kunming 650201, PR China*

[b] *University of Chinese Academy of Sciences, Beijing 100049, PR China*

[c] *School of Life Sciences, Yunnan University, Kunming 650091, China*

[d] *An Shun City People's Hospital, Anshun 561000, China*

[e] *Research Unit of Chemical Biology of Natural Anti-Virus Products, Chinese Academy of Medical Sciences, Beijing 100730, China*

**Corresponding author.*

Ying-Tong Di diyt@mail.kib.ac.cn

Xiao-Jiang Hao haoxj@mail.kib.ac.cn

Chong-Lin Yang clyang@ynu.edu.cn

List of Supporting Information

Biological assays......3
Physical, spectroscopic, and spectrometric data of derivatives 1-19......5
Figure S1. ^{1}H NMR spectra ($CDCl_3$, 500 MHz) of 1......12
Figure S2. ^{13}C NMR spectra ($CDCl_3$, 125 MHz) of 1......13
Figure S3. ^{1}H NMR spectra ($CDCl_3$, 500 MHz) of 2......13
Figure S4. ^{13}C NMR spectra ($CDCl_3$, 125 MHz) of 2......14
Figure S5. ^{1}H NMR spectra ($CDCl_3$, 500 MHz) of 3......14
Figure S6. ^{13}C NMR spectra ($CDCl_3$, 125 MHz) of 3......15
Figure S7. ^{1}H NMR spectra ($CDCl_3$, 500 MHz) of 4......15
Figure S8. ^{13}C NMR spectra ($CDCl_3$, 125 MHz) of 4......16
Figure S9. ^{1}H NMR spectra ($CDCl_3$, 500 MHz) of 5......16
Figure S10. ^{13}C NMR spectra ($CDCl_3$, 125 MHz) of 5......17
Figure S11. ^{1}H NMR spectra ($CDCl_3$, 500 MHz) of 6......17
Figure S12. ^{13}C NMR spectra ($CDCl_3$, 125 MHz) of 6......18
Figure S13. ^{1}H NMR spectra ($CDCl_3$, 500 MHz) of 7......18
Figure S14. ^{13}C NMR spectra ($CDCl_3$, 125 MHz) of 7......19
Figure S15. ^{1}H NMR spectra ($CDCl_3$, 500 MHz) of 8......19
Figure S16. ^{13}C NMR spectra ($CDCl_3$, 125 MHz) of 8......19
Figure S17. ^{1}H NMR spectra ($CDCl_3$, 500 MHz) of 9......20
Figure S18. ^{13}C NMR spectra ($CDCl_3$, 125 MHz) of 9......21
Figure S19. ^{1}H NMR spectra ($CDCl_3$, 500 MHz) of 10......21
Figure S20. ^{13}C NMR spectra ($CDCl_3$, 125 MHz) of 10......22
Figure S21. ^{1}H NMR spectra ($CDCl_3$, 500 MHz) of 11......22
Figure S22. ^{13}C NMR spectra ($CDCl_3$, 125 MHz) of 11......23
Figure S23. ^{1}H NMR spectra ($CDCl_3$, 500 MHz) of 12......23
Figure S24. ^{13}C NMR spectra ($CDCl_3$, 125 MHz) of 12......24
Figure S25. ^{1}H NMR spectra ($CDCl_3$, 500 MHz) of 13......24
Figure S26. ^{13}C NMR spectra ($CDCl_3$, 125 MHz) of 13......24
Figure S27. ^{1}H NMR spectra ($CDCl_3$, 500 MHz) of 14......25
Figure S28. ^{13}C NMR spectra ($CDCl_3$, 125 MHz) of 14......26
Figure S29. ^{1}H NMR spectra ($CDCl_3$, 500 MHz) of 15......26
Figure S30. ^{13}C NMR spectra ($CDCl_3$, 125 MHz) of 15......27
Figure S31. ^{1}H NMR spectra ($CDCl_3$, 500 MHz) of 16......27
Figure S32. ^{13}C NMR spectra ($CDCl_3$, 125 MHz) of 16......27
Figure S33. ^{1}H NMR spectra ($CDCl_3$, 500 MHz) of 17......28
Figure S34. ^{13}C NMR spectra ($CDCl_3$, 125 MHz) of 17......29

Figure S35. ^{1}H NMR spectra ($CDCl_3$, 500 MHz) of 18 29
Figure S36. ^{13}C NMR spectra ($CDCl_3$, 125 MHz) of 18 30
Figure S37. ^{1}H NMR spectra ($CDCl_3$, 500 MHz) of 19 30
Figure S38. ^{13}C NMR spectra ($CDCl_3$, 125 MHz) of 19 30

Biological assays

Cell culture, transfection and reagents:

All cell lines were cultured at 37°C with 5% CO_2 in Dulbeccos modified Eagles medium (DMEM) supplemented with 10% fetal bovine serum (FBS) (HyClone), 100 U ml $^{-1}$ penicillin and 100 mg ml $^{-1}$ streptomycin. No cell lines used in this study were found in the database of commonly misidentified cell lines that is maintained by ICLAC and NCBI Biosample. All cell lines were from ATCC. The cell lines were not authenticated. No mycoplasma contamination of cell lines was found using DAPI staining. Transient transfections were performed with Lipofectamine 2000 (Invitrogen).

Screen for compounds that induce lysosome biogenesis:

HeLa and HepG2 cells with 85% confluency in 24-well plates were treated with individual compounds at 20 μM in triplicate. Three hours later, cells were grown in fresh medium containing Lyso Tracker Red DND-99 (0.3 μM) for 30min.Cells were changed again to Lyso Tracker-free medium and observed by confocal microscopy. Positive candidates were subjected to validation by treating cells with several concentrations in triplicate and staining with Lyso Tracker Red DND-99.

TFEB-EGFP-expressing stable cell lines:

HeLa cells were transfected with pEGFP N_2-TFEB carrying the neomycin (G418)-resistance gene. Forty-eight hours later, cells were further grown in medium supplemented with 5 mg ml $^{-1}$ neomycin for an additional 2-3 weeks. Single colonies were picked up and grown in medium containing neomycin in 96-well plates for one week. Cells were then transferred into 24-well plates for further expansion. Cell lines stably expressing TFEB-EGFP were finally identified by fluorescence microscopy.

Subcellular fractionation:

Cells were lysed in NP-40 buffer (10mM Tris-HCl, pH 7.5, 150mM KCl, 5mM $MgCl_2$ and 0.5% NP-40) for 15 min on ice. The lysates were centrifuged at 1,000 g for 3 min. The supernatant contains the cytosolic and membrane fraction. The pellets were resuspended in NP-40 lysis buffer and sonicated three times for 5s at 20% power to release nuclear proteins.

Lipid droplet clearance assay:

HepG2 cells grown in confocal dishes were fed with oleic acid (100 μM) for 12h to induce lipid formation. Following removal of oleic acid, cells were cultured in fresh medium containing different concentrations of HEP14. Lipid droplets were stained with BODIPY (1μg ml^{-1}) for 0.5 h prior to each time point of examination. Time-course analysis of lipid droplets was performed by observation under a confocal microscope or quantification with flow cytometry.

Lysosome quantification by flow cytometry:

Cells were cultured in medium containing Lyso Tracker Red (0.3 μM) for 30min. After extensive washing, cells were suspended in PBS and transferred into tubes for quantification of lysosome staining by flow cytometry using a FACS AriaII machine (BD Biosciences). Data were analysed using FlowJo software (FLOWJO, LLC).

Western Blot Analysis of Autophagy-Related Proteins:

Human cervical cancer HeLa cells were cultured in DMEM medium supplemented with 10% fetal bovine serum (FBS) under standard conditions (37°C, 95% humidity, 5% CO_2). Cells were seeded in 6-well plates containing growth medium and treated with specified pharmacological agents. Following 24 hours of drug exposure, cells were harvested for subsequent protein extraction and immunoblotting analysis.

Western Blot Analysis:

Cultured HeLa cells were lysed using RIPA buffer (20 mM Tris-HCl, pH 7.5, 100 mM NaCl, 0.1% SDS, 0.5% sodium deoxycholate, and 1 mM PMSF). Protein concentrations were quantified with a BCA Protein Assay Kit BCA Protein Assay Kit (Thermo Fisher Scientific, 23225). Equal amounts of protein (20 μg per lane) were resolved by 12.5% sodium dodecyl sulfate-polyacrylamide gel electrophoresis (SDS-PAGE) and transferred onto nitrocellulose membrane (0.45 μm). Membranes were

blocked with 5% (w/v) non-fat milk in Tris-buffered saline (TBS) for 2 hours at room temperature and subsequently incubated overnight at 4°C with the following primary antibodies: anti-LAMP1 (Proteintech, 21997-1-AP), anti-MAP1LC3B/LC3B (Cell Signaling Technology, 3868), and anti-CTSD (Proteintech, 21327-1-AP), total protein levels were quantified by ponceau S staining (Solarbio, P0012) of the same membrane. After three 3-minute washes with TBST (TBS containing 0.1% Tween-20; Sigma, P1379), membranes were incubated for 1 hour at room temperature with horseradish peroxidase (HRP)-conjugated secondary antibody: Peroxidase-Conjugated Goat anti-Rabbit IgG (H+L) (ZSGB-BIO, ZB-2301), diluted 1:10000 in blocking buffer. Protein bands were visualized using an ECL Western Blot Detection Kit (GE Healthcare and Thermo Fisher Scientific, 35055). Densitometric analysis was performed using ImageJ software (National Institutes of Health), with ponceau S staining serving as the loading control.

Physical, spectroscopic, and spectrometric data of derivatives 1-19

Data for compound 1: Yield: 15 mg, 60%; White powder; ^{1}H NMR ($CDCl_3$, 500 MHz) δ 9.33 (d, J = 1.5 Hz, 1H), 8.80 (dd, J = 4.8, 1.4 Hz, 1H), 8.44 - 8.42 (m, 1H), 7.42 (dd, J = 7.9, 4.9 Hz, 1H), 6.06 (q, J = 7.1 Hz, 1H), 5.92 - 5.91 (d, J = 4.5 Hz, 1H), 5.10 (s, 1H), 4.26 (d, J = 11.7 Hz, 1H), 2.57 - 2.54 (m, 1H), 2.33 (ddd, J = 15.4, 9.6, 2.8 Hz, 1H), 1.87 - 1.84 (m, 6H), 1.78 - 1.72 (m, 4H), 1.57 (s, 3H), 1.12 (s, 3H), 1.06 (s, 3H), 1.00 (d, J = 7.1 Hz, 3H), 0.95 (dd, J = 11.8, 8.4 Hz, 1H), 0.72 - 0.67 (m, 1H); ^{13}C NMR ($CDCl_3$,125 MHz) δ 206.35, 169.17, 165.39, 153.76, 151.46, 139.50, 137.74, 135.48, 134.18, 132.48, 127.22, 126.74, 125.46, 123.27, 86.04, 82.61, 72.27, 43.58, 38.66, 31.13, 28.48, 24.36, 23.21, 23.06, 21.40, 20.66, 17.03, 15.78, 15.66, 15.50; ESIMS *m/z* 520 $[M + Na]^+$; positive ion HRESIMS *m/z* 520.2675 (calcd for $C_{31}H_{37}NO_6Na$ $[M + Na]^+$, 520.2777).

Data for compound 2: Yield: 42 mg, 95%; White powder; ^{1}H NMR ($CDCl_3$, 600 MHz) δ 6.08 (s, 1H), 5.86 - 5.84 (m, 1H), 5.26 (s, 1H), 5.01 (s, 1H), 4.20 - 4.17 (m, 1H), 3.26

(s, 1H), 2.53 - 2.49 (m, 1H), 2.30 - 2.27 (m, 4H), 1.98 - 1.96 (m, 3H), 1.89 (s, 3H), 1.75 (s, 3H), 1.73 - 1.69 (m, 1H),1.56 (s, 3H), 1.05 (d, *J* = 18.7 Hz, 6H), 0.97 (d, *J* = 7.2 Hz, 3H), 0.92 - 0.89 (m, 1H), 0.68 - 0.64 (m, 1H); ^{13}C NMR ($CDCl_3$,150 MHz) *δ* 206.21, 171.02, 169.00, 138.68, 135.27, 134.36, 132.31, 127.51, 126.26, 85.73, 82.15, 71.87, 43.35, 38.67, 31.87, 30.98, 28.41, 24.27, 23.11, 22.93, 21.19, 20.84, 20.71, 16.83, 15.77, 15.53, 15.40; ESIMS *m/z* 479 [M + Na]$^+$; positive ion HRESIMS *m/z* 479.2387 (calcd for $C_{27}H_{36}O_6Na$ [M + Na]$^+$, 479.2380).

Data for compound 3: Yield: 23 mg, 92%; White powder; ^{1}H NMR ($CDCl_3$, 500 MHz) *δ* 6.08 (d, *J* = 1.4 Hz, 1H), 5.87 - 5.80 (m, 1H), 5.21 (s, 1H), 4.91 (s, 1H), 4.21 - 4.13 (m, 1H), 4.47(s, 1H), 2.49 - 2.46 (m, 1H), 2.32 - 2.24 (m, 4H), 2.12 (d, *J* = 1.7 Hz, 3H), 1.76 - 1.70 (m, 5H), 1.54 (s, 3H), 1.24 (s, 3H), 1.07 (s, 3H), 1.04(s, 3H), 0.97 (d, *J* = 7.2 Hz, 3H), 0.90 (dd, *J* = 11.9, 8.4 Hz, 2H), 0.72 - 0.62 (m, 1H); ^{13}C NMR ($CDCl_3$, 125 MHz) *δ* 206.13, 172.49, 135.14, 134.28, 132.60, 126.41, 85.75, 82.65, 77.24, 71.79, 53.43, 43.37, 38.72, 31.10, 29.70, 28.44, 24.35, 23.16, 22.96, 21.20, 20.76, 16.89, 15.56, 15.40; ESIMS *m/z* 417 [M + Na]$^+$; positive ion HRESIMS *m/z* 417.2289 (calcd for $C_{24}H_{32}O_6Na$ [M + Na]$^+$, 417.2286).

Data for compound 4: Yield: 40 mg, 93%; ^{1}H NMR ($CDCl_3$, 500 MHz) *δ* 6.08 (d, *J* = 1.3 Hz, 1H), 5.85 - 5.83 (m, 1H), 5.25 (s, 1H), 4.92 (s, 1H), 4.20 - 4.17 (m, 1H), 2.69 - 2.61 (m, 1H), 2.55 - 2.46 (m, 2H), 2.39 (qd, *J* = 7.6, 2.0 Hz, 2H), 2.32 - 2.27 (m, 1H), 1.75 - 1.70 (m, 4H), 1.54 (s, 3H), 1.21 (t, *J* = 7.5 Hz, 3H), 1.16 (t, *J* = 7.6 Hz, 3H), 1.08 (s, 3H), 1.05 (s, 3H), 0.98 (d, *J* = 7.2 Hz, 3H), 0.91 (dd, *J* = 11.9, 8.4 Hz, 1H), 0.69 - 0.64 (m, 1H); ^{13}C NMR ($CDCl_3$, 125 MHz) *δ* 206.18, 175.73, 174.34, 135.33, 134.39, 132.39, 126.20, 85.74, 82.33, 71.76, 43.32, 38.72, 30.96, 28.40, 27.80, 27.30, 24.29, 23.11, 22.93, 21.20, 16.86, 15.54, 15.39, 9.25, 8.94. ESIMS *m/z* 467 [M + Na]$^+$; positive ion HRESIMS *m/z* 467.1610 (calcd for $C_{26}H_{36}O_6Na$ [M + Na]$^+$, 467.1618).

Data for compound 5: Yield: 18 mg, 90%; ^{1}H NMR ($CDCl_3$, 500 MHz) *δ* 6.08 (d, *J* = 1.3 Hz, 1H), 5.87 - 5.83 (m, 1H), 4.91 (s, 1H), 4.18 (dd, *J* = 11.3, 4.3 Hz, 1H), 2.62 - 2.43 (m, 3H), 2.39 - 2.26 (m, 3H), 1.75 - 1.63 (m, 10H), 1.54 (s, 3H), 1.06 (d, *J* = 16.6 Hz, 6H), 0.99 - 0.95 (m, 9H), 0.93 - 0.89 (m, 1H), 0.69 - 0.64 (m, 1H); ^{13}C NMR ($CDCl_3$,

126 MHz) δ 206.25, 175.02, 173.52, 135.40, 134.44, 132.45, 126.25, 85.81, 82.43, 71.83, 43.39, 38.76, 36.53, 35.81, 30.97, 29.71, 28.45, 24.35, 23.16, 22.97, 21.32, 18.70, 18.10, 16.91, 15.58, 15.43, 13.75, 13.61; ESIMS *m/z* 495 $[M + Na]^+$; positive ion HRESIMS *m/z* 495.1882 (calcd for $C_{28}H_{40}O_6Na$ $[M + Na]^+$, 495.1882).

Data for compound 6: Yield: 101.7 mg, 74%; Yellow oil; ^{1}H NMR ($CDCl_3$, 500 MHz) δ 6.05 (d, *J* = 1.9 Hz, 1H), 5.83 - 5.79 (m, 1H), 5.27 (s, 1H), 4.89 (s, 1H), 4.22 - 4.14 (m, 1H), 2.56 - 2.50 (m, 1H), 2.31 - 2.25 (m, 1H), 1.71 (d, *J* = 1.8 Hz, 3H), 1.73 - 1.67 (m, 1H), 1.51 (s, 3H), 1.29 (s, 9H), 1.18 (s, 9H), 1.07 (s, 3H), 1.03 (s, 3H), 0.96 (d, *J* = 7.2 Hz, 3H), 0.91 - 0.87(m, 1H), 0.67 - 0.62(m, 1H); ^{13}C NMR ($CDCl_3$, 125 MHz) δ 206.31, 179.24, 176.26, 136.02, 134.59, 132.18, 126.06, 86.05, 81.99, 72.13, 43.47, 39.59, 38.91, 38.85, 30.71, 28.47, 27.44, 27.20, 26.53, 24.38, 23.16, 22.99, 21.45, 16.89, 15.69, 15.49, 0.01; ESIMS *m/z* 523 $[M + Na]^+$; positive ion HRESIMS *m/z* 523.3005 (calcd for $C_{30}H_{44}O_6Na$ $[M + Na]^+$, 523.3001).

Data for compound 7: Yield: 28.9 mg, 25%; White powder; ^{1}H NMR ($CDCl_3$, 500 MHz) δ 6.05 (d, *J* = 1.5 Hz, 1H), 5.79 - 5.71 (m, 1H), 5.33 (s, 1H), 4.02 - 3.99 (m, 1H), 3.68 (d, *J* = 6.4 Hz, 1H), 3.33 (s, 1H), 3.05 (d, *J* = 6.8 Hz, 1H), 2.46 - 2.40 (m, 1H), 2.28 - 2.22 (m, 1H), 1.77 - 1.71 (m, 7H), 1.60 (s, 1H), 1.26 - 1.18 (m, 11H), 1.08 (s, 3H), 1.04 (s, 3H), 0.97 (d, *J* = 7.1 Hz, 3H), 0.94 - 0.89 (m, 1H), 0.67 (td, *J* = 8.6, 6.4 Hz, 1H); ^{13}C NMR ($CDCl_3$, 125 MHz) δ 206.86, 179.49, 137.10, 135.31, 132.50, 124.26, 84.91, 83.16, 77.45, 72.01, 43.33, 38.91, 31.07, 28.58, 27.22, 23.93, 23.31, 23.20, 21.98, 17.21, 15.54; ESIMS *m/z* 439 $[M + Na]^+$; positive ion HRESIMS *m/z* 439.2452 (calcd for $C_{25}H_{36}O_5Na$ $[M + Na]^+$, 439.2455).

Data for compound 8: Yield: 3.1 mg, 44%; White powder; ^{1}H NMR ($CDCl_3$, 500 MHz) δ 5.96 (d, *J* = 1.5 Hz, 1H), 5.69 (d, *J* = 1.5 Hz, 1H), 5.15 (s, 1H), 4.90 (ddd, *J* = 49.0, 11.2, 9.0 Hz, 1H), 3.06 (dd, *J* = 16.8, 9.0 Hz, 1H), 2.24 - 2.19 (m, 1H), 1.88 (s, 3H), 1.68 (d, *J* = 1.4 Hz, 3H), 1.59 - 1.56 (m, 2H), 1.24 (s, 9H), 1.17 (d, *J* = 7.1 Hz, 3H), 1.10 (s, 3H), 1.08 (s, 3H), 0.87 - 0.86 (m, 1H), 0.75 - 0.69 (m, 1H); ^{13}C NMR ($CDCl_3$, 125 MHz) δ 206.92, 179.43, 141.98, 138.79, 133.80, 128.99, 96.12, 94.70, 85.99, 80.05, 64.99, 63.21, 63.02, 43.08, 39.08, 29.04, 28.44, 27.18, 24.10, 23.69, 22.61, 20.87, 15.06,

14.11; ESIMS *m/z* 419 [M + H]$^+$; positive ion HRESIMS *m/z* 419.2521 (calcd for $C_{25}H_{36}FO_4$ [M + H]$^+$, 419.2519).

Data for compound 9: Yield: 104 mg, 93%; White solid; ^{1}H NMR ($CDCl_3$, 500 MHz) δ 5.83 (d, *J* = 5.9 Hz, 2H), 4.68 (s, 1H), 4.02 (dd, *J* = 10.9, 5.3 Hz, 1H), 3.78 (d, *J* = 6.4 Hz, 1H), 2.66 - 2.60 (m, 1H), 2.46 (d, *J* = 7.6 Hz, 1H), 2.15 (ddd, *J* = 15.7, 7.0, 2.6 Hz, 1H), 1.87 - 1.79 (m, 6H), 1.73 - 1.67 (m, 1H), 1.60 (s, 3H), 1.52 (d, *J* = 13.9 Hz, 6H), 1.14 (s, 3H), 1.03 - 0.96 (m, 8H), 0.67 (dd, *J* = 15.1, 6.8 Hz, 1H); ^{13}C NMR ($CDCl_3$, 125 MHz) δ 207.41, 137.52, 136.83, 130.65, 125.98, 113.79, 96.27, 89.56, 75.72, 74.77, 44.13, 38.63, 31.62, 28.67, 27.89, 27.00, 23.84, 23.39, 23.27, 23.04, 17.99, 15.37, 15.26; ESIMS *m/z* 395 [M + Na]$^+$; positive ion HRESIMS *m/z* 395.2198 (calcd for $C_{23}H_{32}O_4Na$ [M + Na]$^+$, 395.2193).

Data for compound 10: Yield: 90 mg, 85%; White power; ^{1}H NMR ($CDCl_3$, 500 MHz) δ 5.76 (d, *J* = 1.2 Hz, 1H), 5.37 (dd, *J* = 5.4, 1.3 Hz, 1H), 4.28 (d, *J* = 1.1 Hz, 1H), 4.11 (dt, *J* = 7.1, 6.3 Hz, 1H), 3.99 (s, 1H), 3.23 (s, 3H), 2.64 - 2.63 (m, 1H), 2.04 (s, 2H), 1.87 (d, *J* = 1.4 Hz, 3H), 1.70 - 1.56 (m, 4H), 1.44 (s, 3H), 1.37 (d, *J* = 2.0 Hz, 6H), 1.35 - 1.22 (m, 4H), 1.05 (s, 3H), 0.96 (s, 3H), 0.80 (d, *J* = 6.6 Hz, 3H), 0.73 (t, *J* = 8.5 Hz, 1H), 0.48 (dd, *J* = 9.3, 4.7 Hz, 1H); ^{13}C NMR ($CDCl_3$, 125 MHz) δ 151.10, 137.42, 134.54, 131.41, 112.75, 94.17, 88.57, 84.46, 75.87, 74.09, 60.41, 50.86, 36.25, 35.88, 29.11, 27.32, 26.08, 25.25, 25.00, 23.98, 19.41, 17.62, 17.51, 17.31, 15.08, 14.21. ESIMS *m/z* 427 [M + Na]$^+$; positive ion HRESIMS *m/z* 427.2453 (calcd for $C_{24}H_{36}O_5Na$ [M + Na]$^+$, 427.2455).

Data for compound 11: Yield: 5.9 mg, 59 %; Yellow oil; ^{1}H NMR ($CDCl_3$, 500 MHz) δ 5.87-5.85 (m, 1H), 5.73 (s, 1H), 5.30 (s, 1H), 4.34 (s, 1H), 4.19 (s, 1H), 2.79 - 2.74 (m,1H), 2.48 - 2.36 (m, 3H), 2.14 - 2.10 (m, 1H), 1.82 - 1.81 (m, 4H), 1.75 (s, 3H), 1.59 (s, 3H), 1.43 (m, 7H), 1.21 - 1.17 (m, 8H), 1.08 - 1.02 (m, 4H), 0.99 (d, *J* = 6.8 Hz, 4H), 0.72 - 0.68 (m, 1H); ^{13}C NMR ($CDCl_3$, 125 MHz) δ 207.40, 173.61, 137.46, 135.82, 129.61, 128.10, 113.27, 94.74, 88.39, 76.45, 75.79, 44.12, 37.22, 32.52, 28.71, 28.03, 27.61, 27.15, 24.91, 23.75, 22.97, 22.92, 18.93, 15.51, 14.82, 9.18; ESIMS *m/z* 451 [M

+ Na]$^+$; positive ion HRESIMS *m/z* 451.2455 (calcd for $C_{26}H_{36}O_5Na$ [M + Na]$^+$, 451.2455).

Data for compound 12： Yield: 8.2 mg, 55%, Yellow oil; ^{1}H NMR ($CDCl_3$, 500 MHz) δ 5.86 (dd, J = 6.0, 1.4 Hz, 1H), 5.72 (s, 1H), 5.30 (d, J = 2.1 Hz, 1H), 4.35(s,1H), 4.21 - 4.20 (m, 1H), 3.48 (s,1H), 2.78 - 2.75 (m, 1H), 2.46 - 2.29 (m, 2H) , 2.12 (ddd, J = 15.9, 4.7, 2.8 Hz, 1H), 1.82 (s, 3H), 1.75 - 1.55 (m, 10H), 1.43 (s, 6H), 1.34 - 1.31 (m, 5H), 1.22 (s, 3H), 1.08 - 1.03 (m, 4H), 0.99 (d, J = 6.8 Hz, 3H), 0.93 - 0.87 (m, 4H), 0.70 (dt, J = 8.8, 5.7 Hz, 1H); ^{13}C NMR ($CDCl_3$, 125 MHz) δ 207.49, 172.93, 135.88, 129.47, 128.21, 113.22, 94.69, 88.38, 77.28, 77.02, 76.77, 75.87, 44.08, 37.10, 34.31, 32.62, 31.28, 29.71, 28.72, 28.15, 27.20, 24.99, 24.65, 23.81, 23.07, 22.88, 22.33, 18.98, 15.54, 14.77, 13.92; ESIMS *m/z* 493 [M + Na]$^+$; positive ion HRESIMS *m/z* 493.2922 (calcd for $C_{29}H_{42}O_5Na$ [M + Na]$^+$, 493.2924).

Data for compound 13： Yield: 40 mg, 32%; White solid; ^{1}H NMR ($CDCl_3$, 500 MHz) δ 5.88 (dd, J = 5.8, 1.5 Hz, 1H), 5.63 (s, 1H), 5.30 (s, 1H), 4.37 (s, 1H), 4.24 - 4.21 (m, 1H), 2.83 - 2.79 (m, 1H), 2.04 (dt, J = 15.8, 3.1 Hz, 1H), 1.79 (d, J = 14.6 Hz, 6H), 1.76 - 1.69 (m, 1H), 1.42 - 1.38 (m, 6H), 1.27 - 1.19 (m, 16H), 1.10 (dd, J = 10.7, 9.0 Hz, 1H), 1.01 - 0.96 (m, 6H), 0.70 – 0.66 (m, 1H); ^{13}C NMR ($CDCl_3$, 125 MHz) δ 208.32, 177.83, 138.27, 135.96, 128.90, 128.59, 112.79, 94.49, 88.36, 77.13, 76.05, 43.56, 38.98, 36.69, 32.97, 28.67, 28.63, 27.42, 27.30, 27.01, 26.52, 25.14, 24.43, 23.57, 22.60, 19.18, 16.15, 14.55; ESIMS *m/z* 495 [M + K]$^+$; positive ion HRESIMS *m/z* 495.2869 (calcd for $C_{28}H_{40}O_5K$ [M + K]$^+$, 495.2507).

Data for compound 14： Yield: 28mg, 23%; White power; ^{1}H NMR ($CDCl_3$, 500 MHz) δ 5.89 (d, J = 5.4 Hz, 1H), 5.69 (s, 1H), 5.41 (s, 1H), 4.91 (d, J = 9.4 Hz, 1H), 4.43 (s, 1H), 4.30 (dd, J = 9.4, 4.0 Hz, 1H), 4.14 (d, J = 10.3 Hz, 1H), 3.71 (s, 5H), 2.75 – 2.72 (m, 1H), 2.10 (d, J = 16.1 Hz, 1H), 1.93 - 1.90 (m, 1H), 1.79 (s, 3H), 1.76 - 1.67 (m, 5H), 1.45 - 1.41 (m, 17H), 1.24 - 1.21 (m, 4H), 1.09 - 1.02 (m, 6H), 0.98 (d, J = 6.7 Hz, 6H), 0.87 - 0.84 (m, 3H), 0.71 - 0.67 (m, 1H); ^{13}C NMR ($CDCl_3$, 125 MHz) δ 207.90, 172.20, 155.85, 137.77, 135.18, 129.26, 128.71, 113.50, 94.44, 88.43, 79.92, 75.81,

63.71, 58.70, 43.99, 37.26, 36.94, 32.47, 29.70, 28.70, 28.60, 28.28, 27.97, 27.12, 24.77, 23.92, 23.65, 23.04, 22.92, 18.99, 16.25, 15.72, 14.74, 11.55; ESIMS *m/z* 608 [M + Na]$^+$; positive ion HRESIMS *m/z* 608.4370 (calcd for $C_{34}H_{51}NO_7Na$ [M + Na]$^+$, 608.4370).

Data for compound 15： Yield: 6 mg, 18%; White powder; Transparent oil; ^{1}H NMR ($CDCl_3$, 500 MHz) δ 6.10 (s, 1H), 5.78 (d, J = 4.4 Hz, 1H), 5.30 (s, 1H), 5.01 (d, J = 9.4 Hz, 1H), 4.30 (dd, J = 9.5, 5.0 Hz, 1H), 4.02 (dd, J = 11.6, 3.5 Hz, 1H), 2.67 - 2.66 (m, 1H), 2.29 - 2.23 (m, 1H), 2.11 - 2.06 (m, 1H), 1.82 - 1.77 (m, 8H), 1.64 - 1.58 (m, 20H), 1.46 - 1.43 (d, J = 8.9 Hz, 13H), 1.32 (s, 3H), 1.25 (s, 3H), 1.11 (s, 3H), 1.06 (s, 3H), 0.97 (dd, J = 6.9, 3.9 Hz, 6H), 0.91 - 0.86 (m, 5H), 0.72 - 0.67 (m, 1H); ^{13}C NMR ($CDCl_3$, 125 MHz) δ 205.64, 175.17, 156.71, 134.24, 133.07, 131.48, 126.27, 85.99, 83.27, 79.80, 71.76, 63.71, 52.96, 52.48, 43.39, 42.16, 39.94, 30.82, 29.70, 28.39, 28.33, 28.29, 24.83, 24.72, 24.29, 23.32, 23.06, 22.90, 21.96, 21.18, 16.92, 15.47, 15.41; ESIMS *m/z* 572 [M + H]$^+$; positive ion HRESIMS *m/z* 572.4425 (calcd for $C_{33}H_{50}NO_7$ [M + H]$^+$, 572.4425).

Data for compound 16: Yield: 15 mg, 9%; White powder; ^{1}H NMR ($CDCl_3$, 500 MHz) δ 5.92 (d, J = 6.4 Hz, 1H), 5.53 (s, 1H), 5.31 (s, 1H), 5.16 (d, J = 7.7 Hz, 1H), 4.46 (s, 3H), 2.88 (s, 1H), 2.62 (t, J = 7.4 Hz, 2H), 2.25 - 2.20 (dd, m, 1H), 2.14 - 2.00 (m, 5H), 1.85 - 1.83 (m, J = 12.7 Hz, 6H), 1.79 - 1.72 (m, 1H), 1.62 (s, 2H), 1.44 (s, 9H), 1.36 - 1.25 (m, 12H), 1.16 (t, J = 9.6 Hz, 1H), 1.00 - 0.97 (m, 7H), 0.89 - 0.82 (m, 2H), 0.73 - 0.70 (m, 1H); ^{13}C NMR ($CDCl_3$, 125 MHz) δ 207.67, 171.71, 155.13, 139.07, 135.20, 131.08, 127.62, 112.45, 93.76, 88.27, 79.96, 71.81, 53.00, 43.67, 35.51, 33.91, 33.22, 29.81, 29.71, 29.37, 28.79, 28.31, 27.70, 26.32, 24.30, 24.03, 22.32, 19.83, 19.17, 15.68, 15.34, 14.13, 13.94; ESIMS *m/z* 626 [M + Na]$^+$; positive ion HRESIMS *m/z* 626.3923 (calcd for $C_{33}H_{49}NO_7SNa$ [M + Na]$^+$, 626.3919).

Data for compound 17: Yield: 33 mg, 29%; Light yellow power; ^{1}H NMR ($CDCl_3$, 500 MHz) δ 5.88 (d, J = 5.2 Hz, 1H), 5.80 (s, 1H), 5.36 (s, 1H), 4.80 (d, J = 8.8 Hz, 1H), 4.46 (s, 1H), 4.36 - 4.32 (m, 1H), 43.99 - 3.96 (m, 1H), 3.73 (s, 1H), 2.70 - 2.68

(m, 1H), 2.16 (dd, *J* = 15.3, 4.1 Hz, 1H), 1.80 (s, 3H), 1.76 - 1. 73(m, 2H), 1.69 - 1.67 (m, 5H), 1.50 - 1.43 (m, 17H), 1.19 (s, 3H), 1.03 (s, 3H), 0.98 (d, *J* = 6.8 Hz, 3H), 0.94 - 0.91 (m, 6H), 0.71 - 0.67 (m, 1H); ^{13}C NMR ($CDCl_3$, 125 MHz) *δ* 207.57, 173.23, 155.60, 150.93, 137.13, 134.92, 130.19, 128.48, 127.56, 113.88, 94.96, 88.31, 79.95, 76.35, 75.38, 68.23, 63.72, 52.45, 44.33, 40.53, 38.14, 31.85, 28.61, 28.30, 27.45, 26.85, 24.71, 23.58, 23.31, 22.55, 21.14, 18.54, 15.86, 15.20; ESIMS *m/z* 608 $[M + Na]^+$; positive ion HRESIMS *m/z* 608.4370 (calcd for $C_{34}H_{51}NO_7Na$ $[M + Na]^+$, 608.4374).

Data for compound 18: Yield: 18 mg, 13%; White power; ^{1}H NMR ($CDCl_3$, 500 MHz) *δ* 5.89 (d, *J* = 4.8 Hz, 1H), 5.73 (s, 1H), 5.37 (s, 1H), 5.29 (s, 1H), 5.10 (d, *J* = 8.2 Hz, 1H), 4.42 (s, 1H), 4.38 - 5.34 (m, 1H), 4.13 - 4.09 (m, 1H), 3.72 (s, 1H), 3.67 (s, 4H), 2.73 - 2.70 (m, 1H), 2.43 - 2.40 (m, 2H), 2.23 (ddd, *J* = 19.2, 9.5, 5.9 Hz, 1H), 2.14 - 2.10 (m, 1H), 1.93 - 1.86(m, 1H), 1.80 - 1.70 (m, 1H), 1.46 - 1.43 (m, 21H), 1.26 - 1.23 (mz, 1H), 1.20 - 1.19 (m, 4H), 1.06 - 1.02 (m, 6H), 0.98 (d, *J* = 6.8 Hz, 4H), 0.69 (dt, *J* = 8.6, 6.0 Hz, 1H); ^{13}C NMR ($CDCl_3$, 125 MHz) *δ* 207.52, 173.10, 171.89, 155.49, 137.51, 134.81, 129.60, 128.57, 113.64, 94.59, 88.38, 80.19, 75.68, 63.71, 53.38, 51.88, 44.14, 37.56, 32.28, 30.18, 28.63, 28.27, 27.88, 27.02, 24.54, 23.75, 23.00, 22.89, 18.82, 15.57, 14.90, 14.20, 14.12; ESIMS *m/z* 616 $[M + H]^+$; positive ion HRESIMS *m/z* 616.3472 (calcd for $C_{34}H_{50}NO_9$ $[M + H]^+$, 616.3472).

Data for compound 19: Yield: 7.8 mg, 7%; Transparent oil; ^{1}H NMR ($CDCl_3$, 600 MHz) *δ* 7.33 - 7.28 (m, 3H), 7.22 (d, *J* = 7.1 Hz, 2H), 5.92 (d, *J* = 5.5 Hz, 1H), 5.58 (s, 1H), 5.38 (s, 1H), 4.98 (d, *J* = 8.7 Hz, 1H), 4.62 (d, *J* = 5.6 Hz, 1H), 4.40 (s, 1H), 4.27 (s, 1H), 3.74 (s,6H), 3.33 - 3.30 (m, *J* = 13.7, 4.6 Hz, 1H), 2.94 - 2.91 (m, *J* = 13.7, 7.8 Hz, 2H), 2.09 - 2.04 (m, 3H), 1.92 (d, *J* = 35.3 Hz, 4H), 1.83 (s, 3H), 1.81 (s, 2H),1.62 (s, 3H), 1.39 - 1.25 (m, 29H), 1.02 - 0.98 (m, 10H), 0.89 - 0.84 (m, 2H), 0.72 (s, 1H); ^{13}C NMR ($CDCl_3$, 150 MHz) *δ* 207.73, 171.33, 154.87, 136.14, 135.23, 130.91, 129.49, 128.83, 128.53, 127.03, 112.75, 93.92, 88.23, 79.86, 71.79, 63.69, 62.18, 54.73, 43.74, 38.80, 35.97, 33.61, 29.68, 28.96, 28.68, 28.23, 27.94, 27.57, 25.93, 24.18, 23.80, 22.40, 20.81, 19.64, 19.14, 15.62, 14.14; ESIMS *m/z* 620 $[M + H]^+$; positive ion HRESIMS *m/z* 620.4309 (calcd for $C_{37}H_{50}NO_7$ $[M + H]^+$, 620.4317).

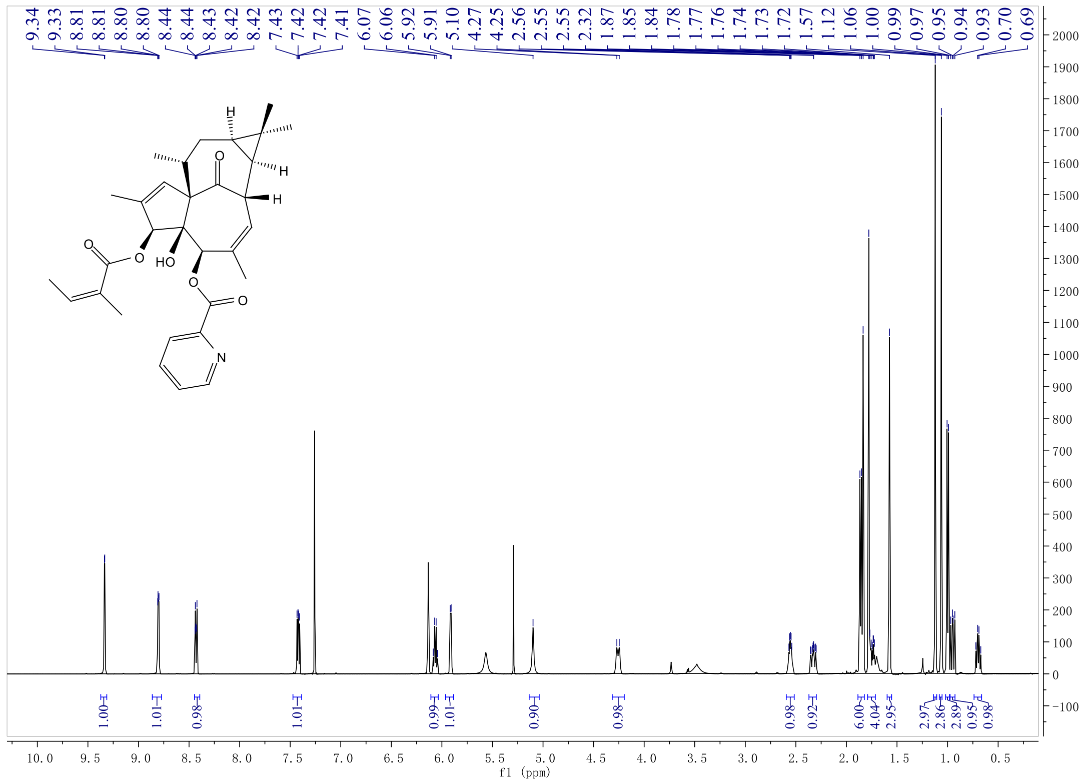

Figure S1. ^{1}H NMR spectra ($CDCl_3$, 500 MHz) of **1**

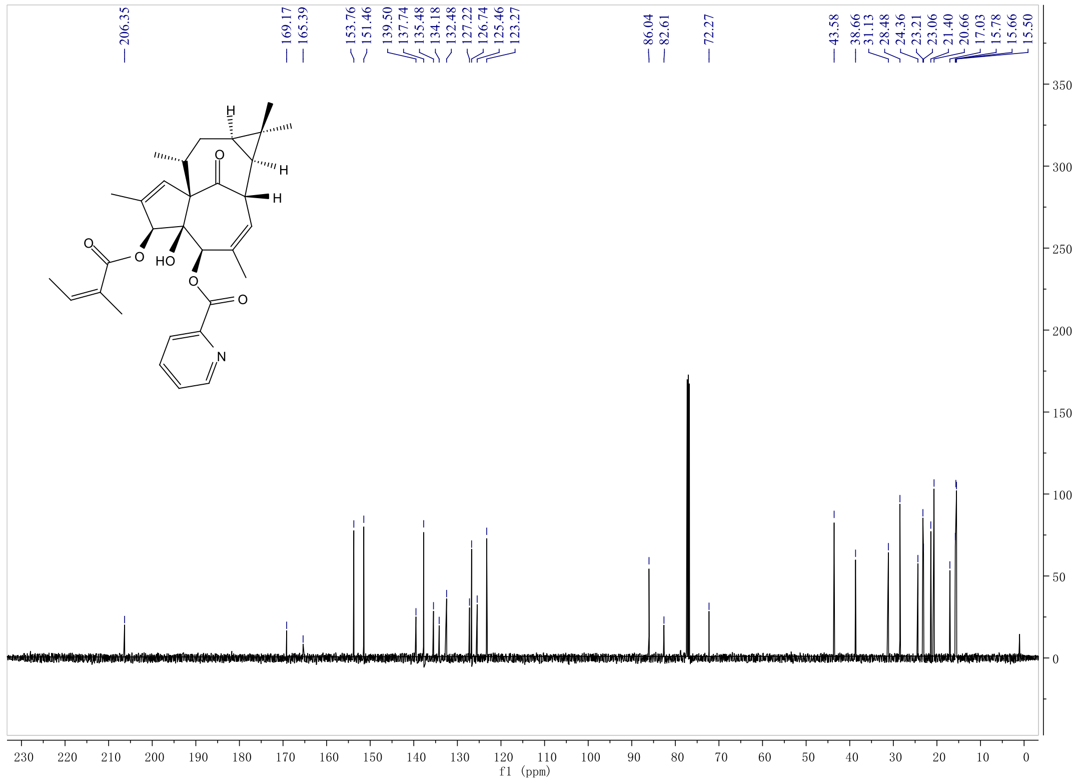

Figure S2. ^{13}C NMR spectra (CDCl$_3$, 125 MHz) of **1**

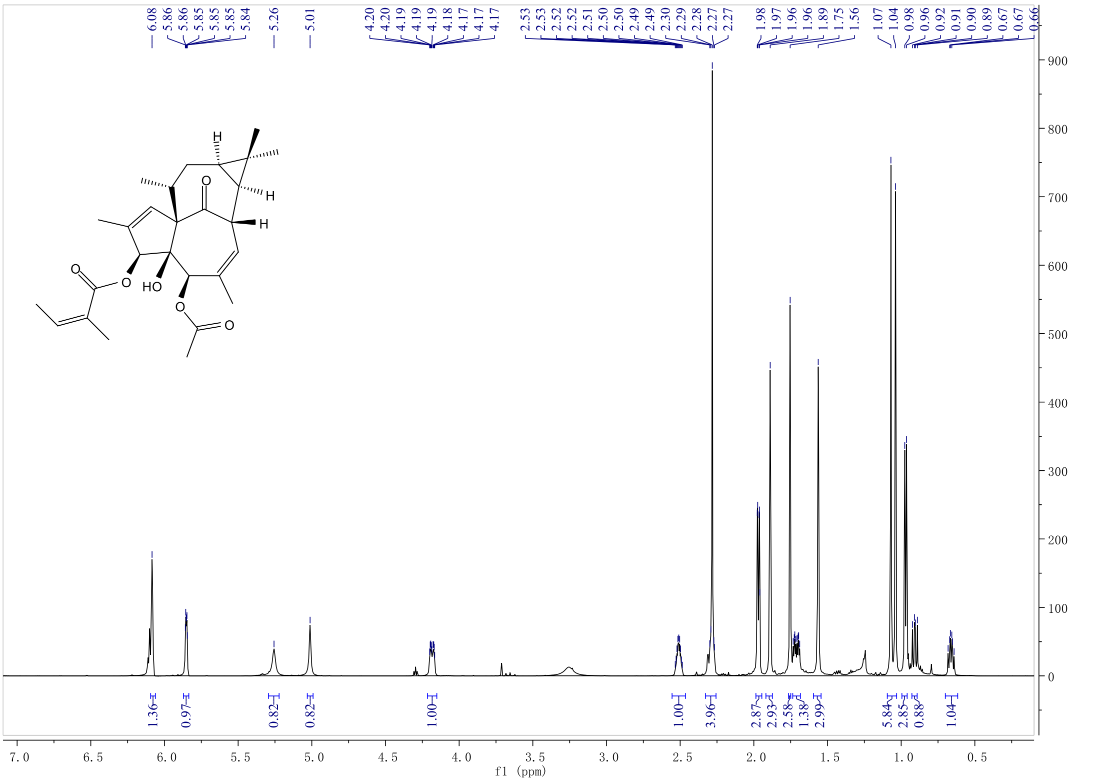

Figure S3. ^{1}H NMR spectra (CDCl$_3$, 500 MHz) of **2**

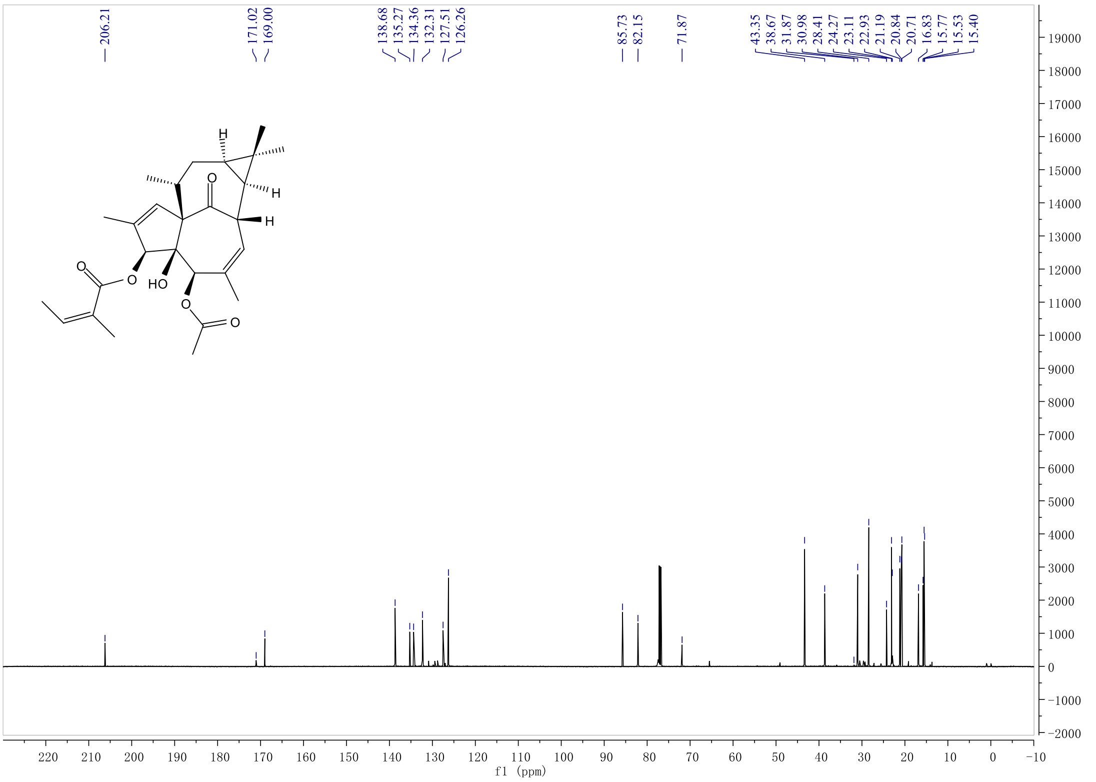

Figure S4. ^{13}C NMR spectra ($CDCl_3$, 125 MHz) of **2**

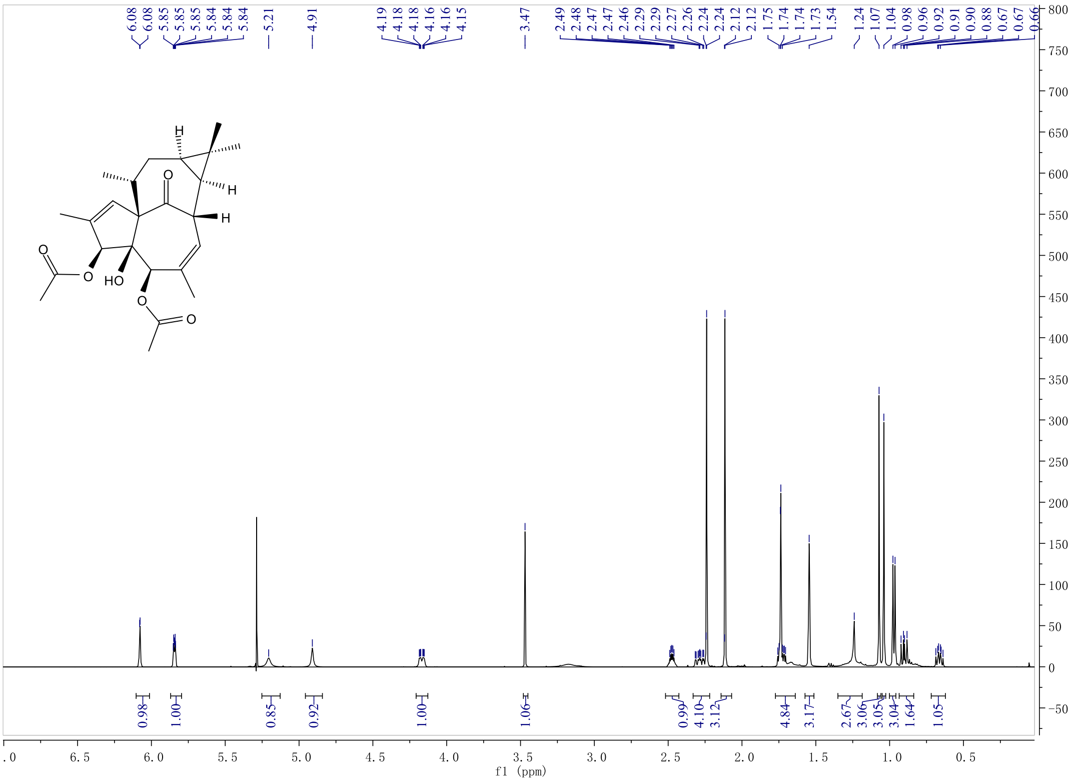

Figure S5. ^{1}H NMR spectra ($CDCl_3$, 500 MHz) of **3**

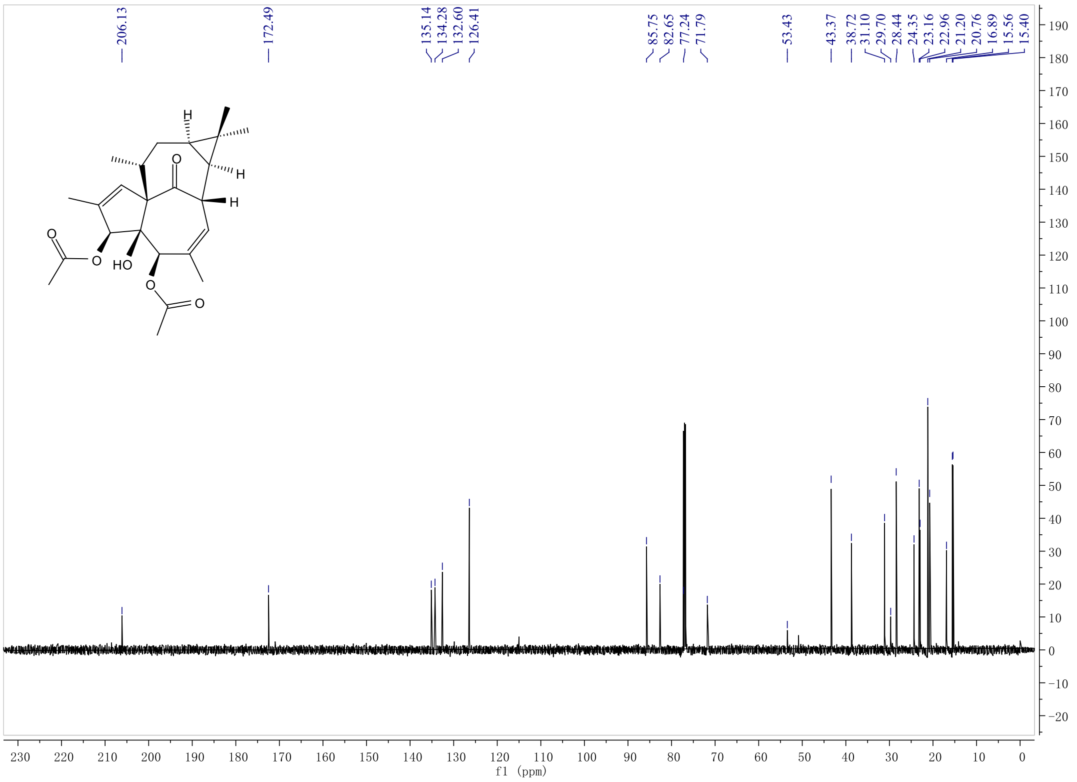

Figure S6. ^{13}C NMR spectra ($CDCl_3$, 125 MHz) of **3**

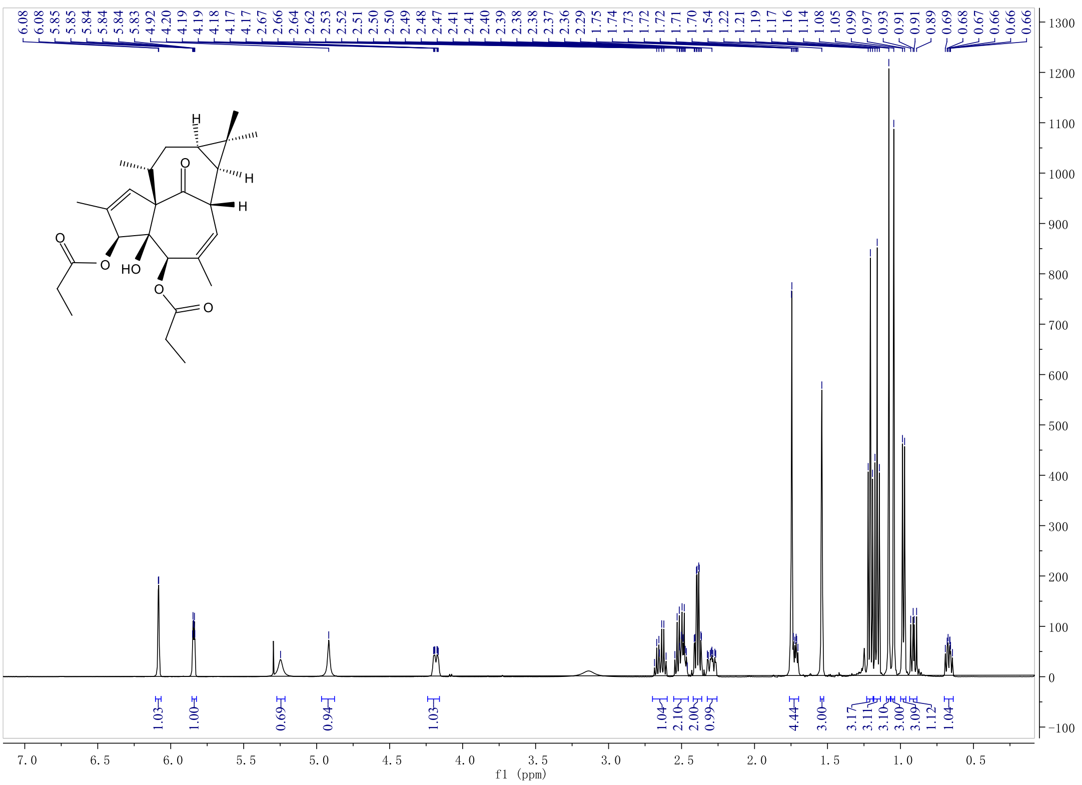

Figure S7. ^{1}H NMR spectra ($CDCl_3$, 500 MHz) of **4**

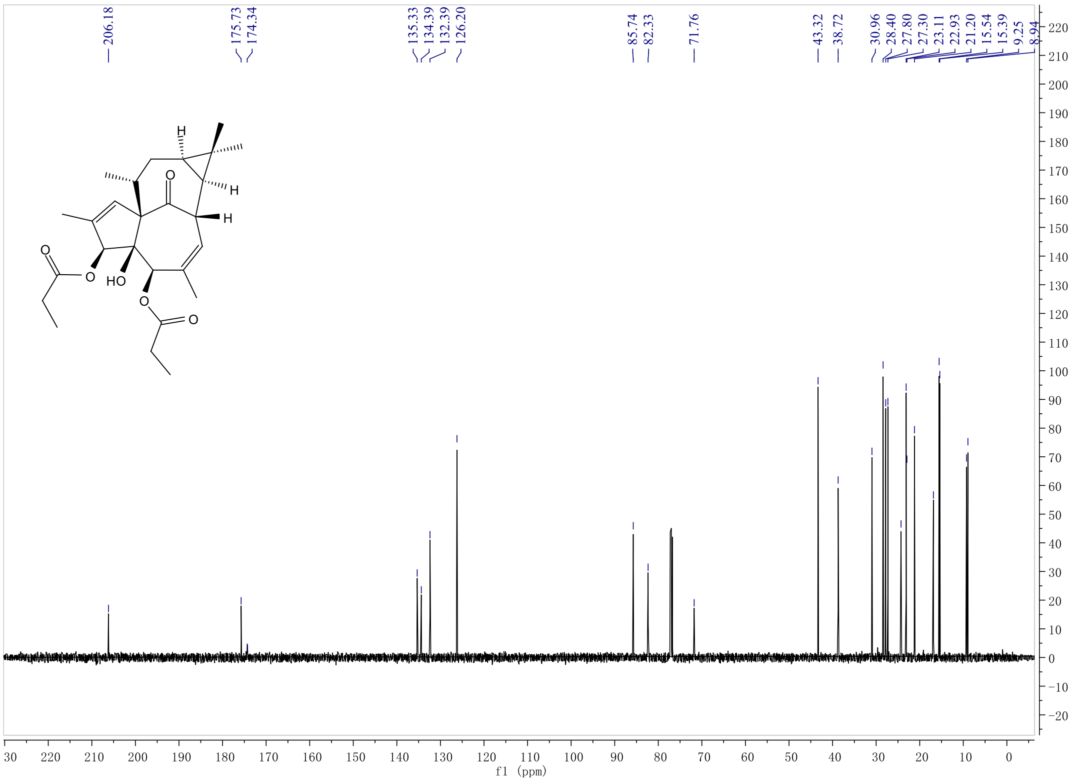

Figure S8. ^{13}C NMR spectra ($CDCl_3$, 125 MHz) of **4**

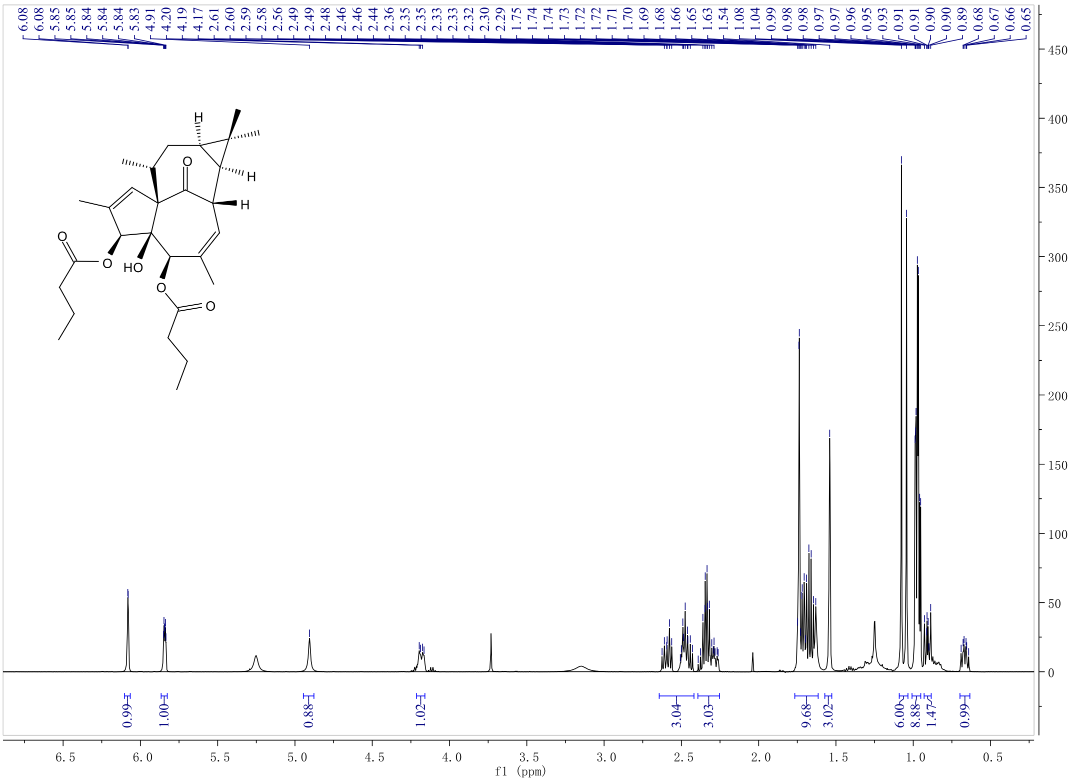

Figure S9. ^{1}H NMR spectra ($CDCl_3$, 500 MHz) of **5**

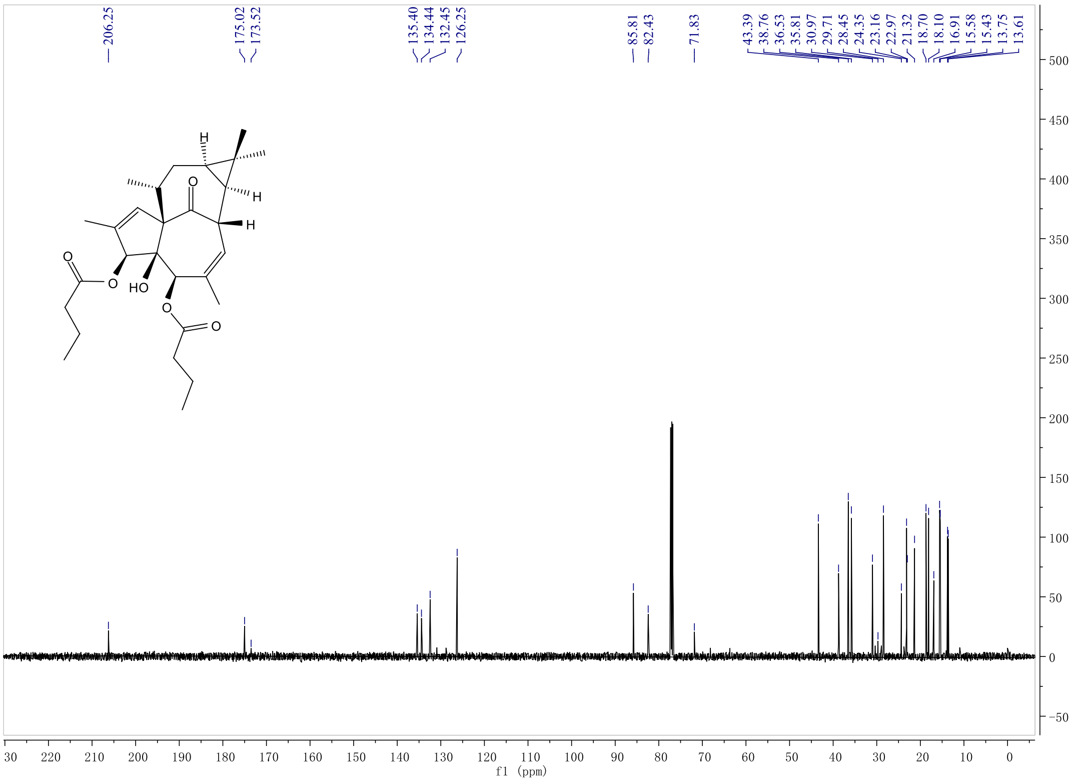

Figure S10. ^{13}C NMR spectra (CDCl$_3$, 125 MHz) of **5**

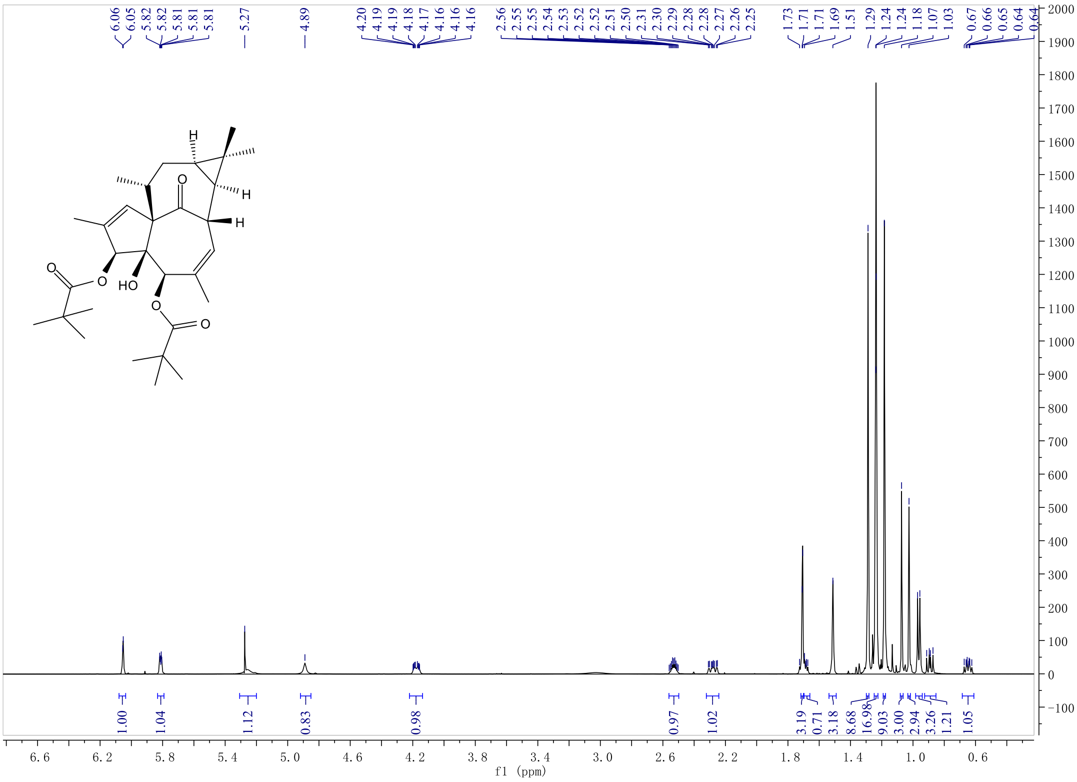

Figure S11. ^{1}H NMR spectra (CDCl$_3$, 500 MHz) of **6**

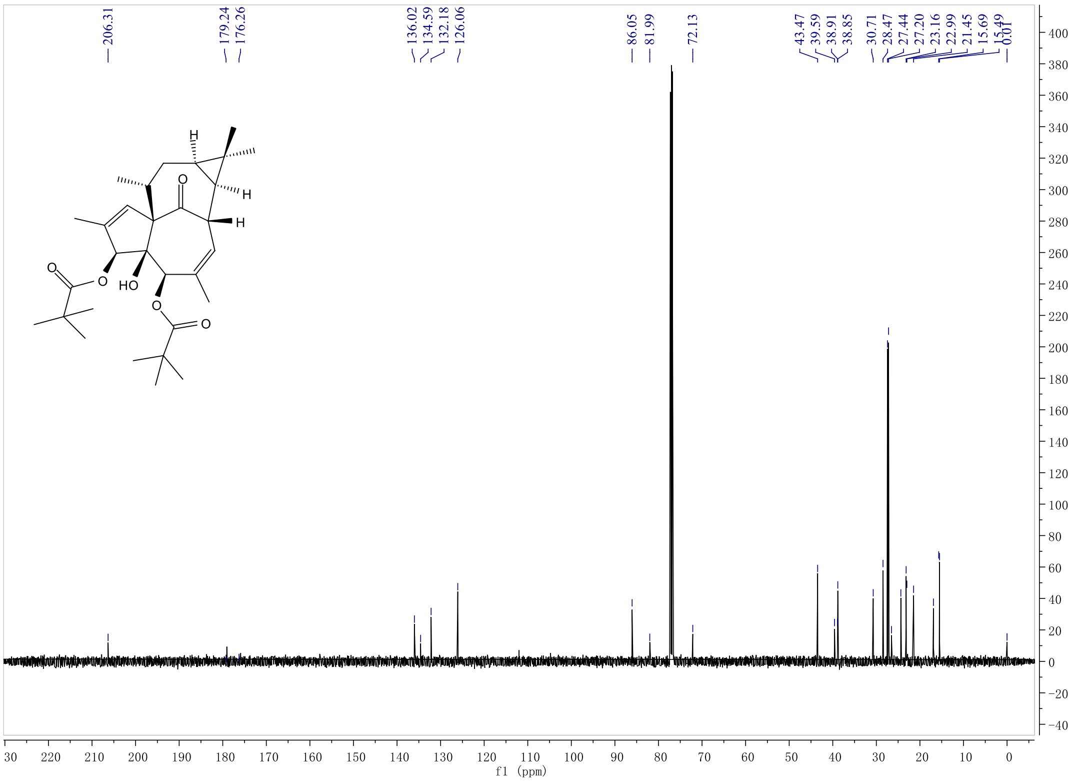

Figure S12. ^{13}C NMR spectra ($CDCl_3$, 125 MHz) of **6**

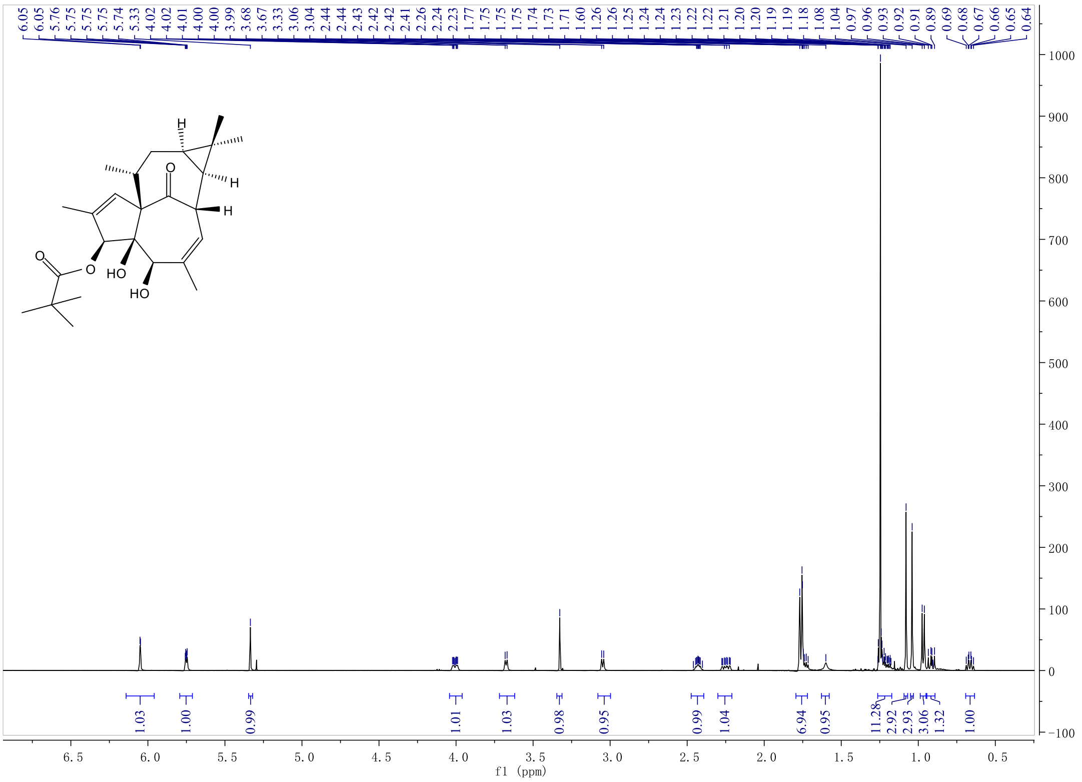

Figure S13. ^{1}H NMR spectra ($CDCl_3$, 500 MHz) of **7**

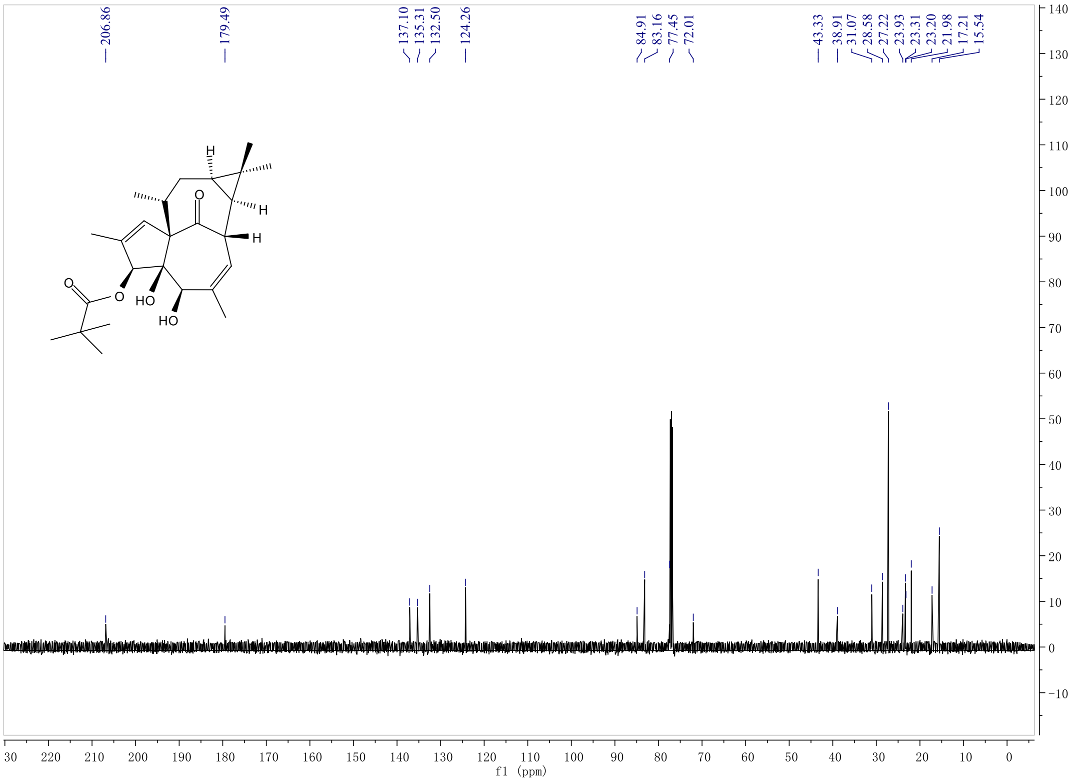

Figure S14. ^{13}C NMR spectra (CDCl$_3$, 125 MHz) of **7**

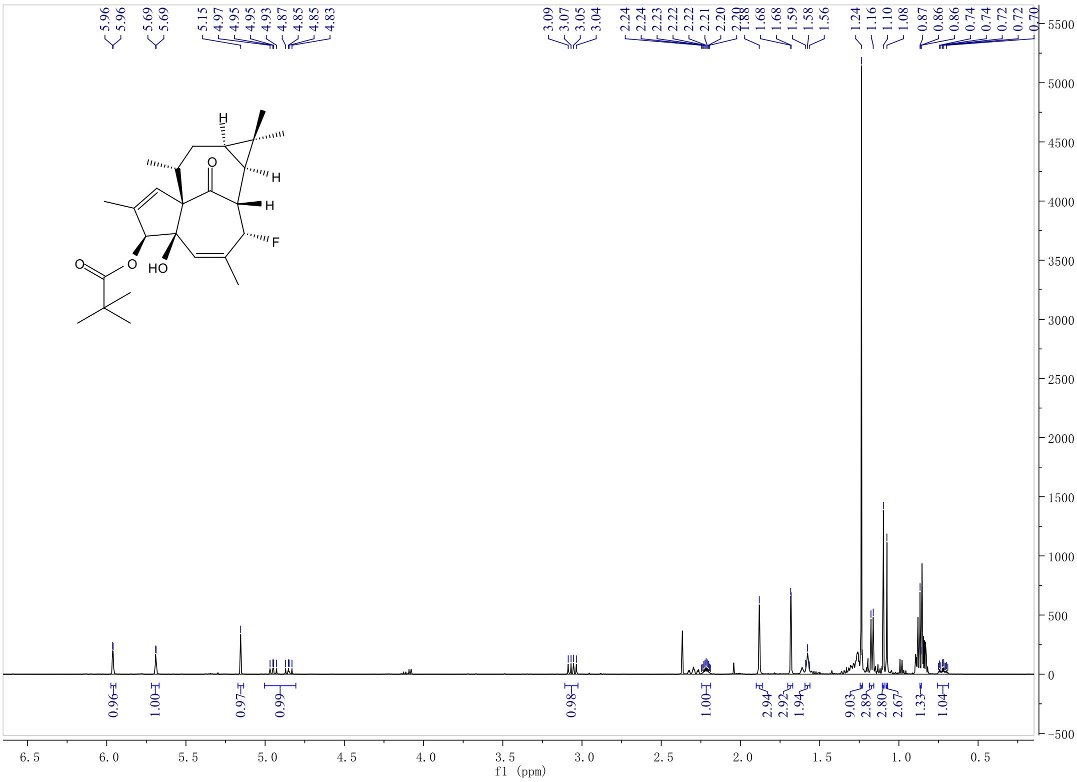

Figure S15. ^{1}H NMR spectra (CDCl$_3$, 500 MHz) of **8**

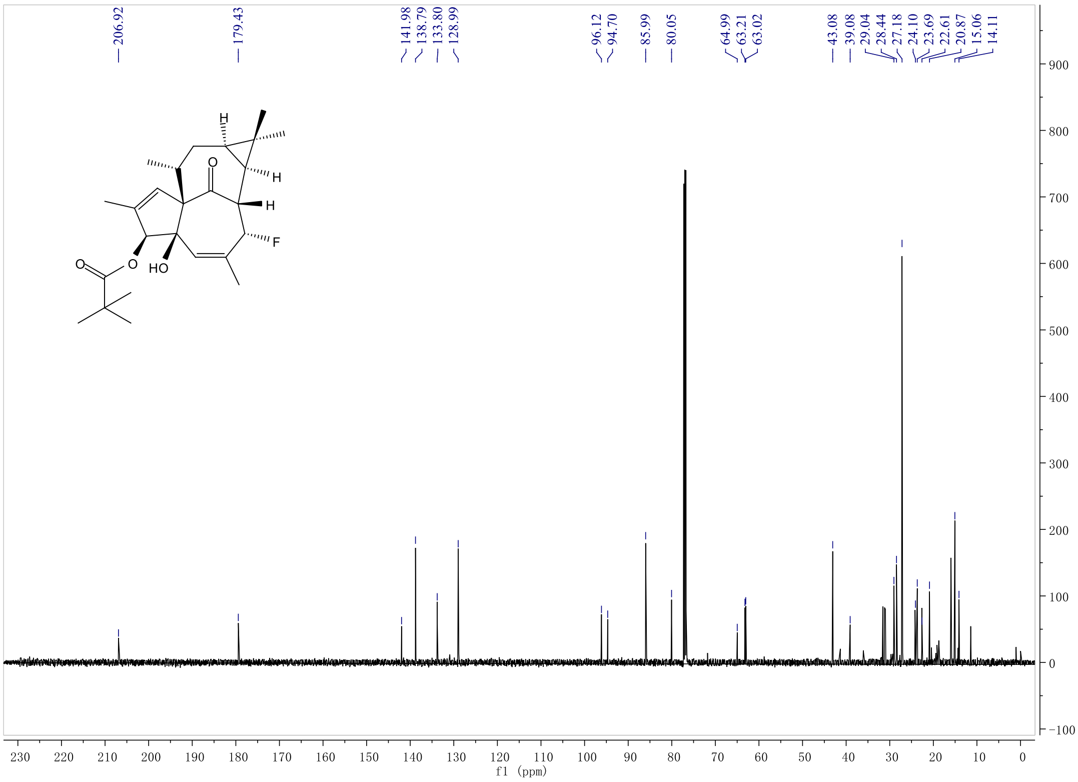

Figure S16. ^{13}C NMR spectra (CDCl$_3$, 125 MHz) of **8**

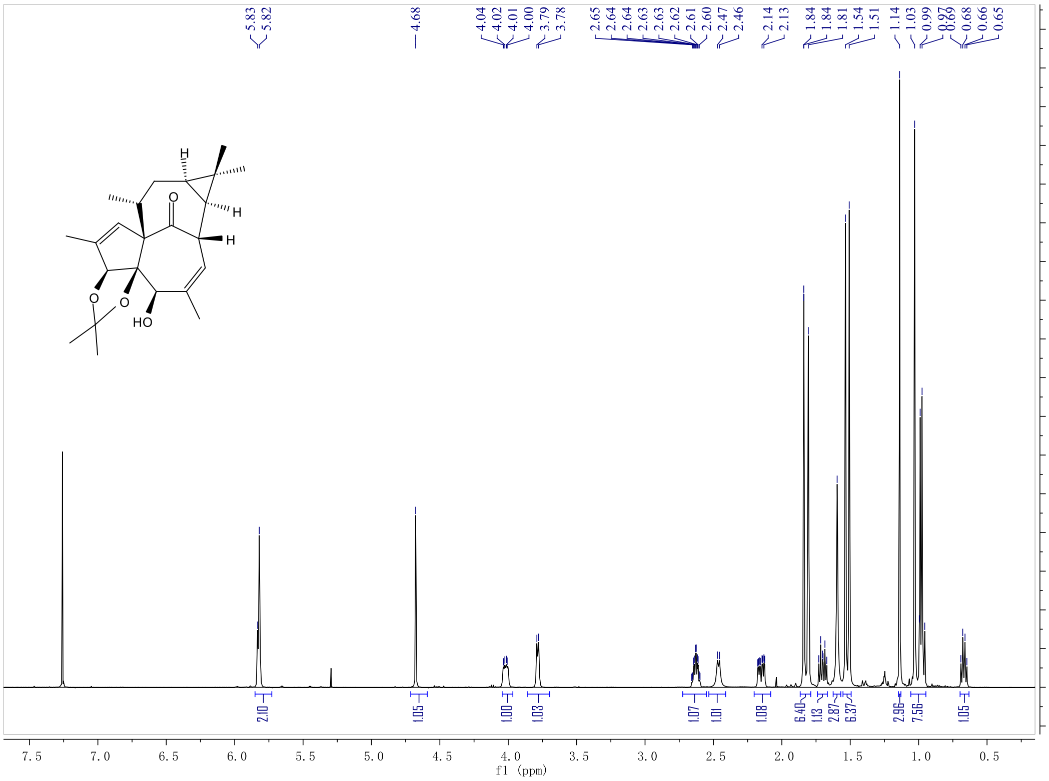

Figure S17. ^{1}H NMR spectra ($CDCl_3$, 500 MHz) of **9**

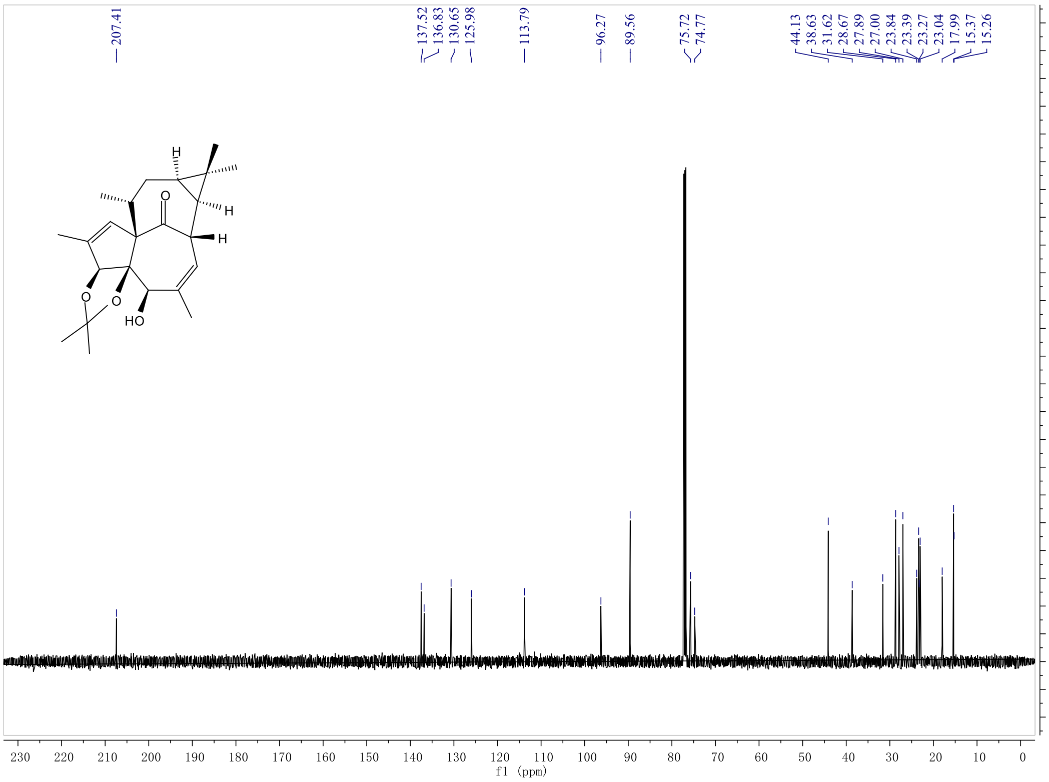

Figure S18. ^{13}C NMR spectra ($CDCl_3$, 125 MHz) of **9**

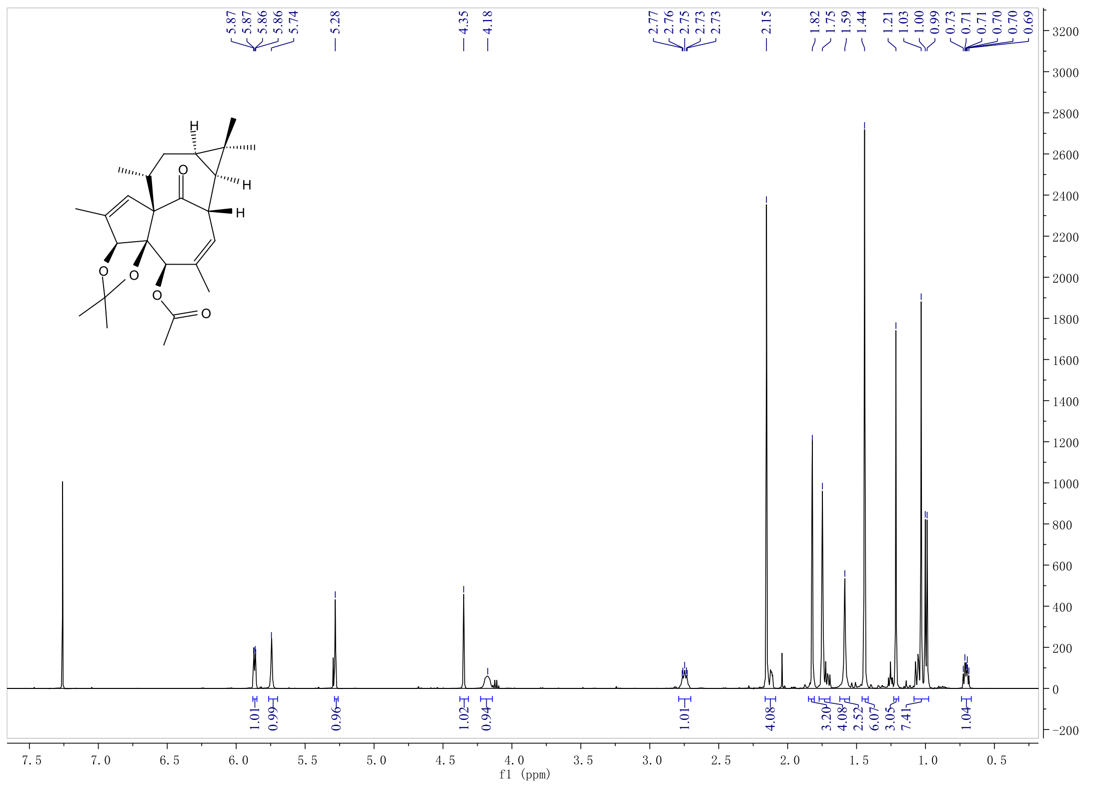

Figure S19. ^{1}H NMR spectra ($CDCl_3$, 500 MHz) of **10**

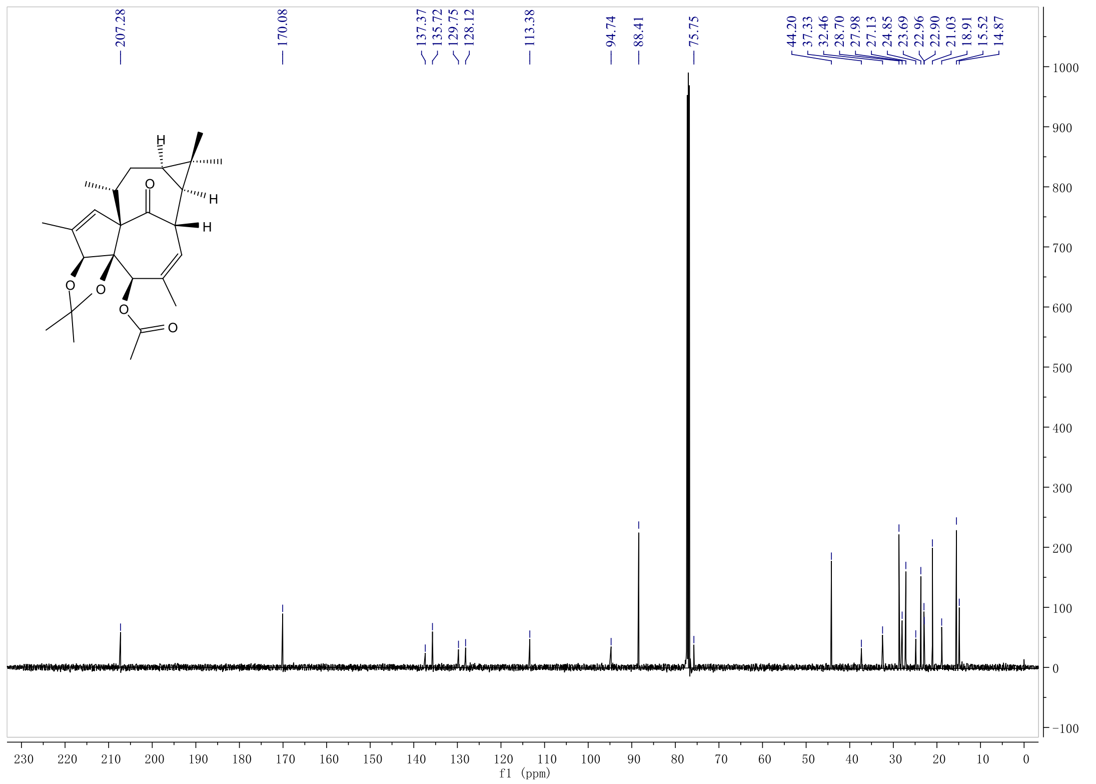

Figure S20. ^{13}C NMR spectra ($CDCl_3$, 125 MHz) of **10**

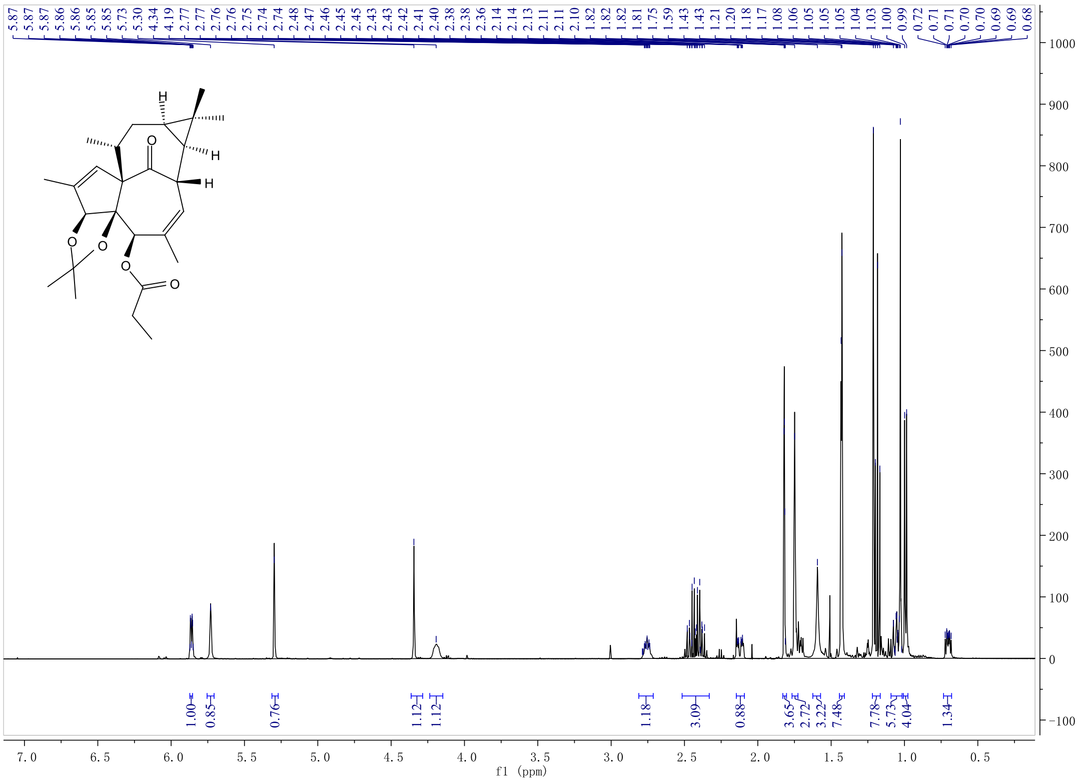

Figure S21. ^{1}H NMR spectra ($CDCl_3$, 500 MHz) of **11**

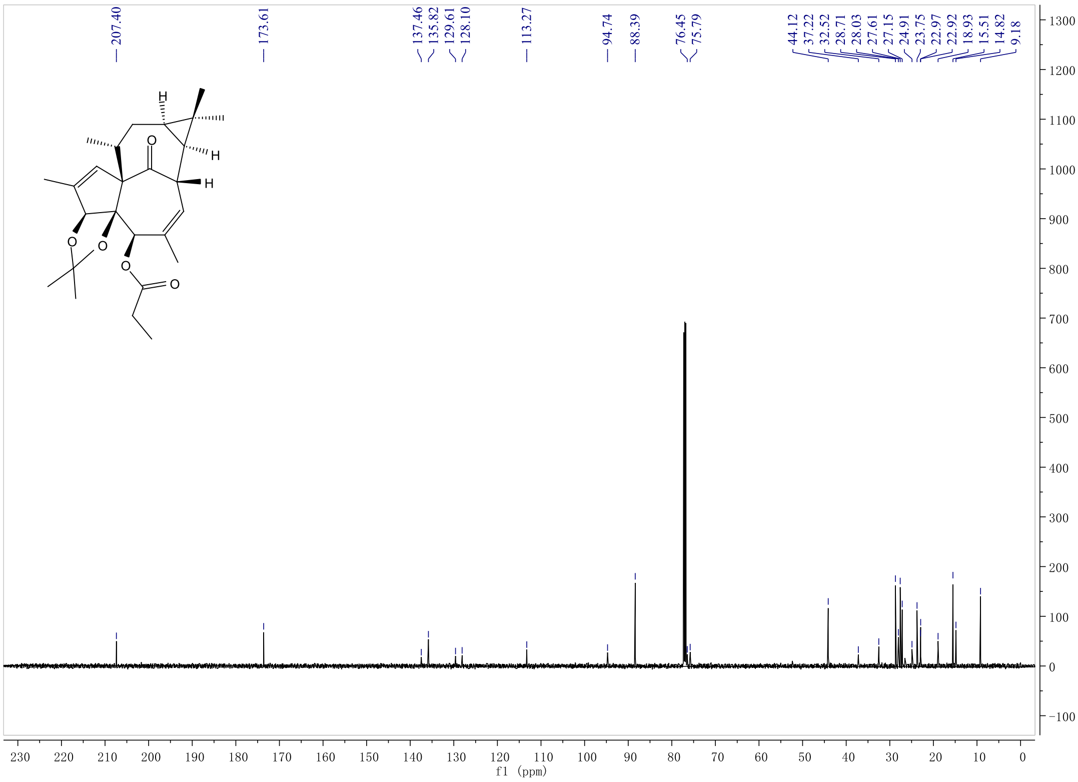

Figure S22. ^{13}C NMR spectra ($CDCl_3$, 125 MHz) of **11**

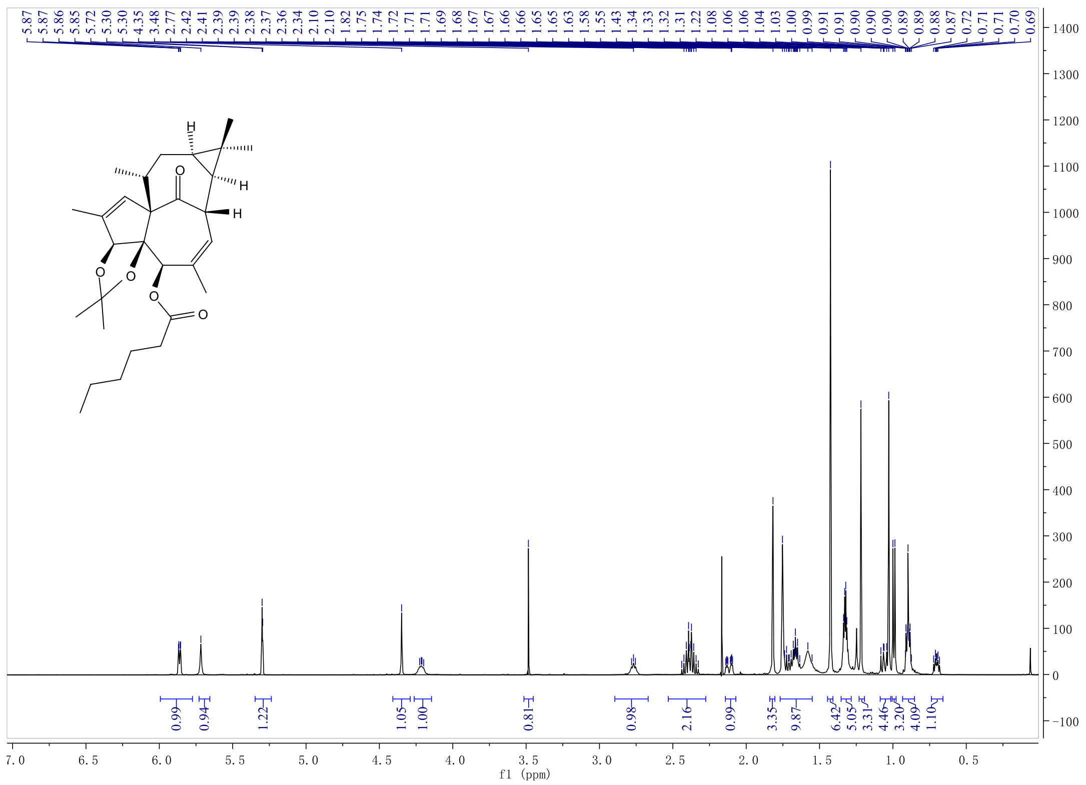

Figure S23. ^{1}H NMR spectra ($CDCl_3$, 500 MHz) of **12**

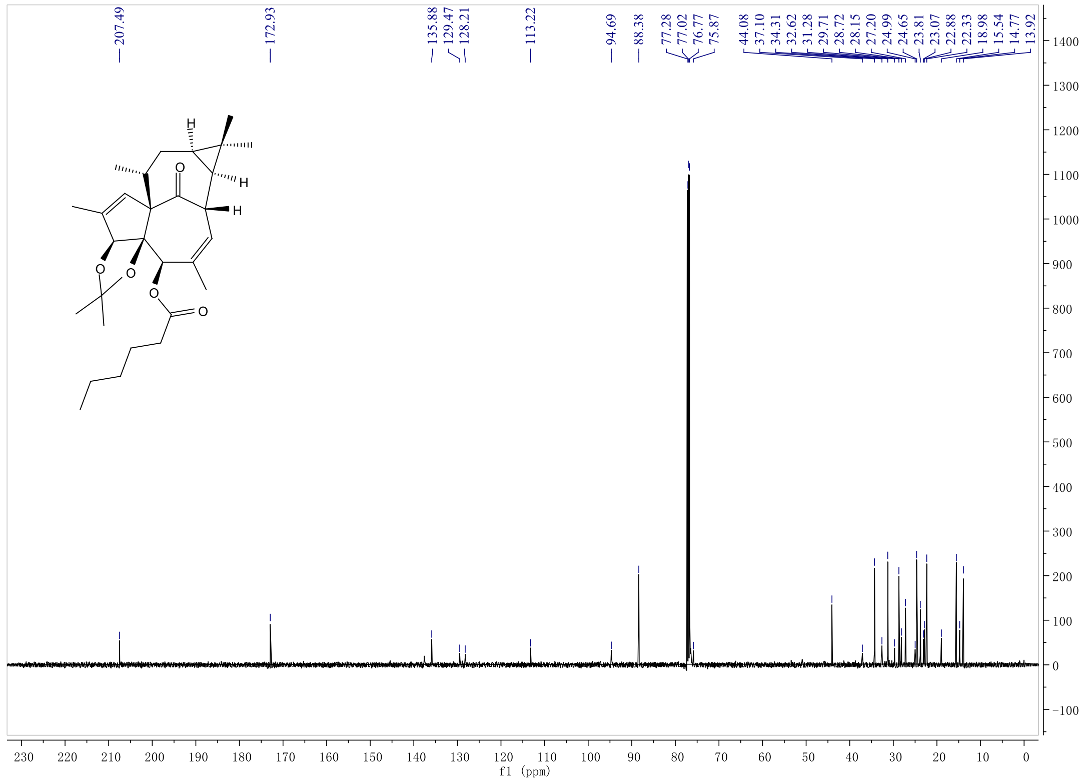

Figure S24. ^{13}C NMR spectra ($CDCl_3$, 125 MHz) of **12**

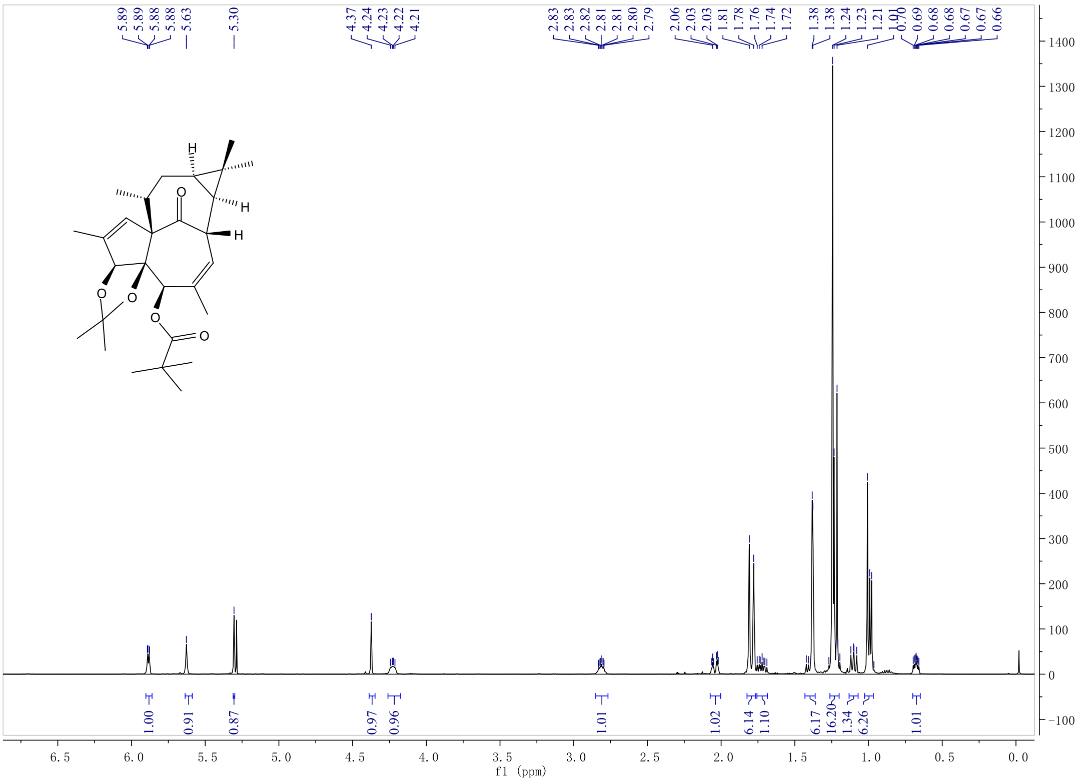

Figure S25. ^{1}H NMR spectra ($CDCl_3$, 500 MHz) of **13**

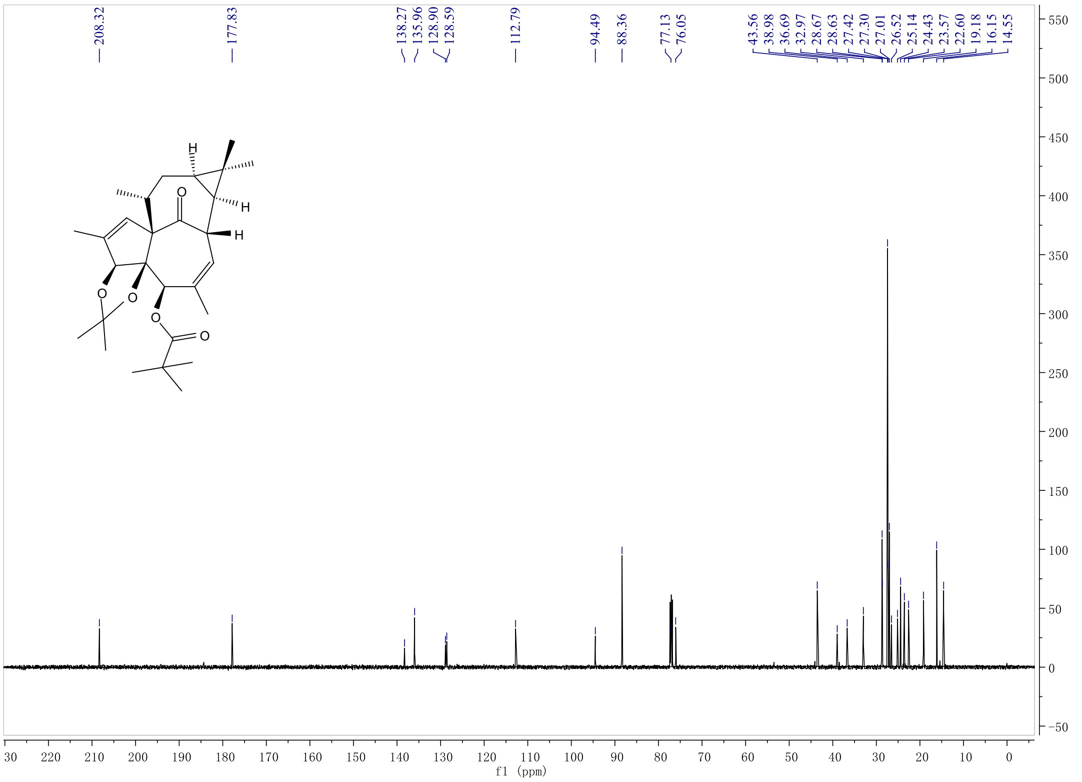

Figure S26. ^{13}C NMR spectra ($CDCl_3$, 125 MHz) of **13**

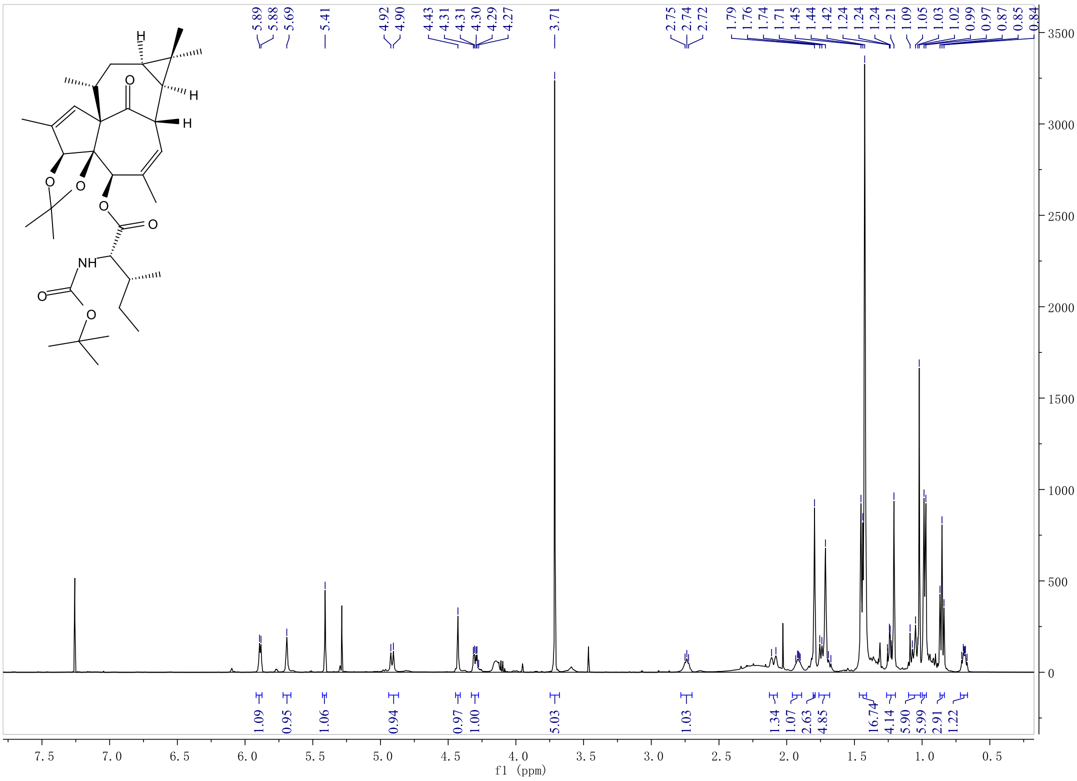

Figure S27. ^{1}H NMR spectra ($CDCl_3$, 500 MHz) of **14**

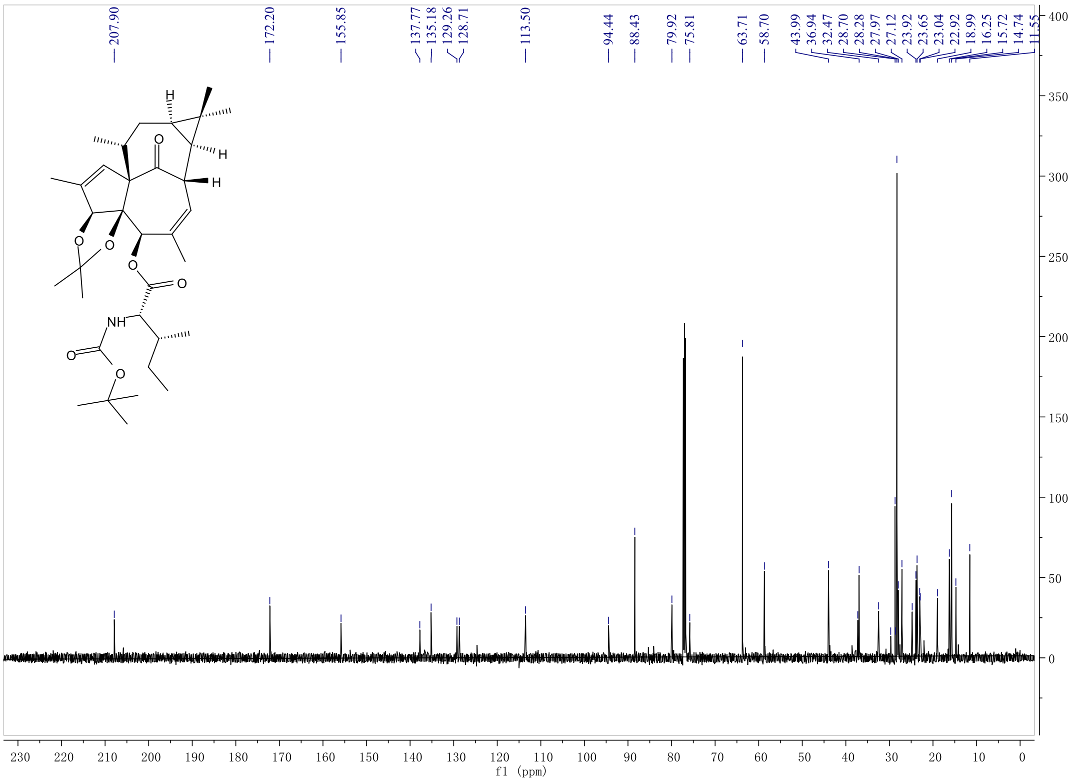

Figure S28. ^{13}C NMR spectra ($CDCl_3$, 125 MHz) of **14**

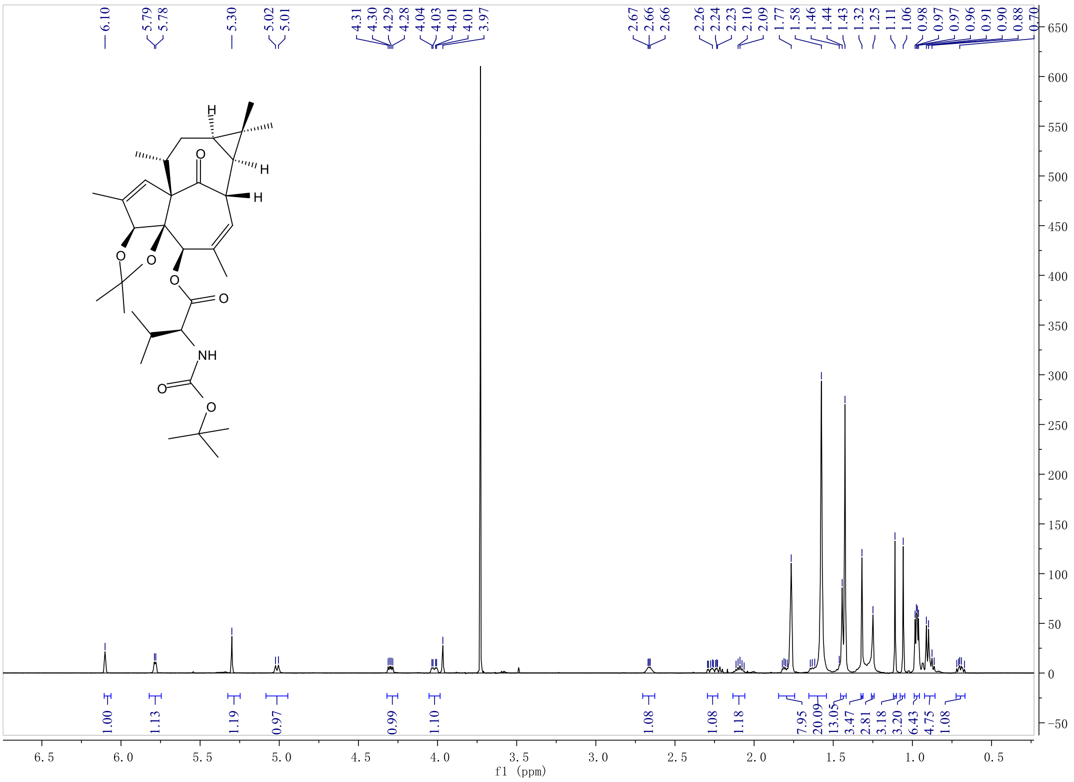

Figure S29. ^{1}H NMR spectra ($CDCl_3$, 500 MHz) of **15**

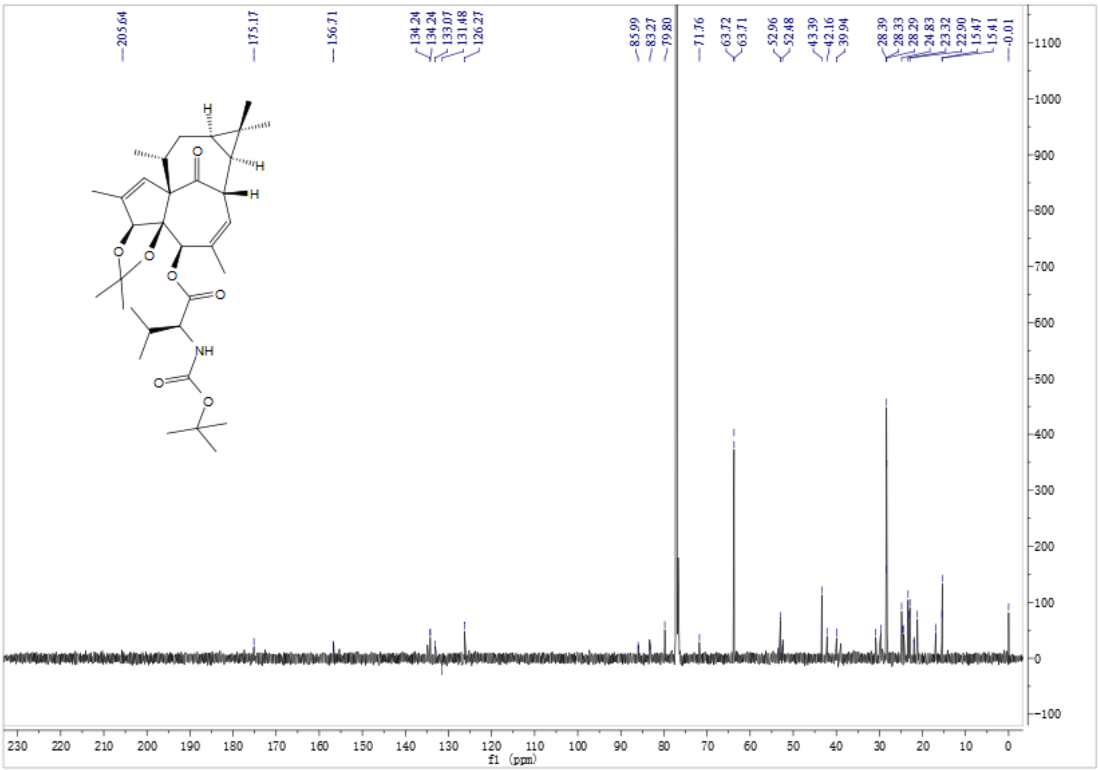

Figure S30. ^{13}C NMR spectra ($CDCl_3$, 125 MHz) of **15**

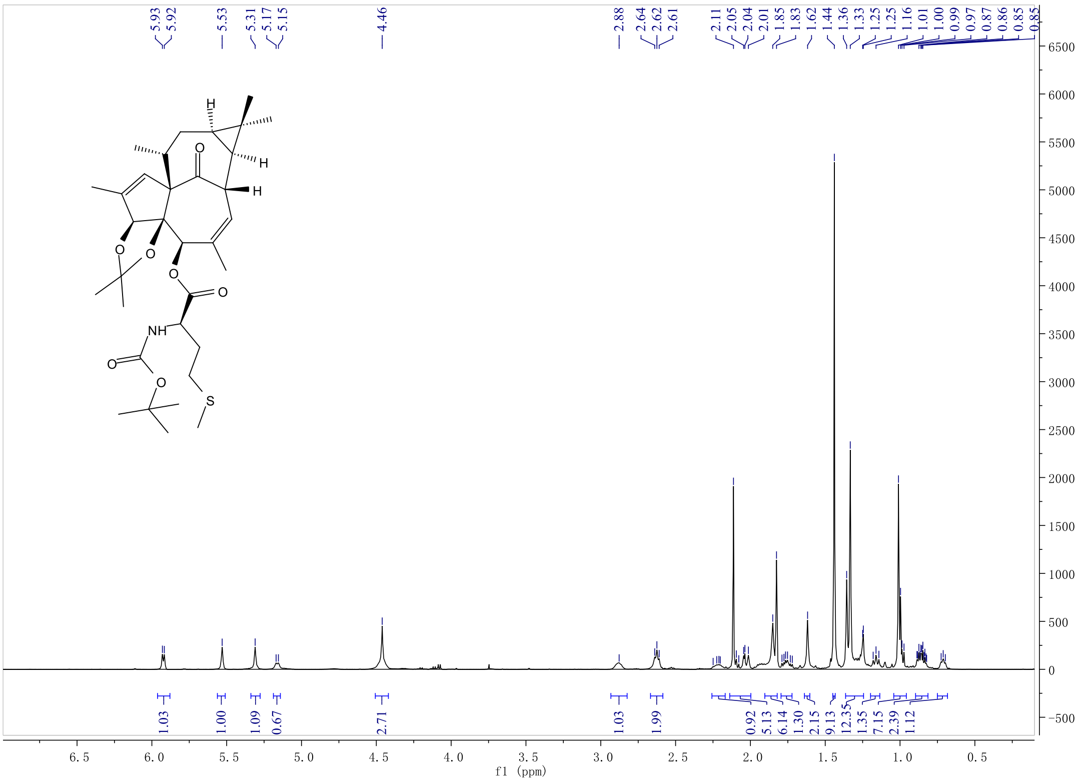

Figure S31. ^{1}H NMR spectra ($CDCl_3$, 500 MHz) of **16**

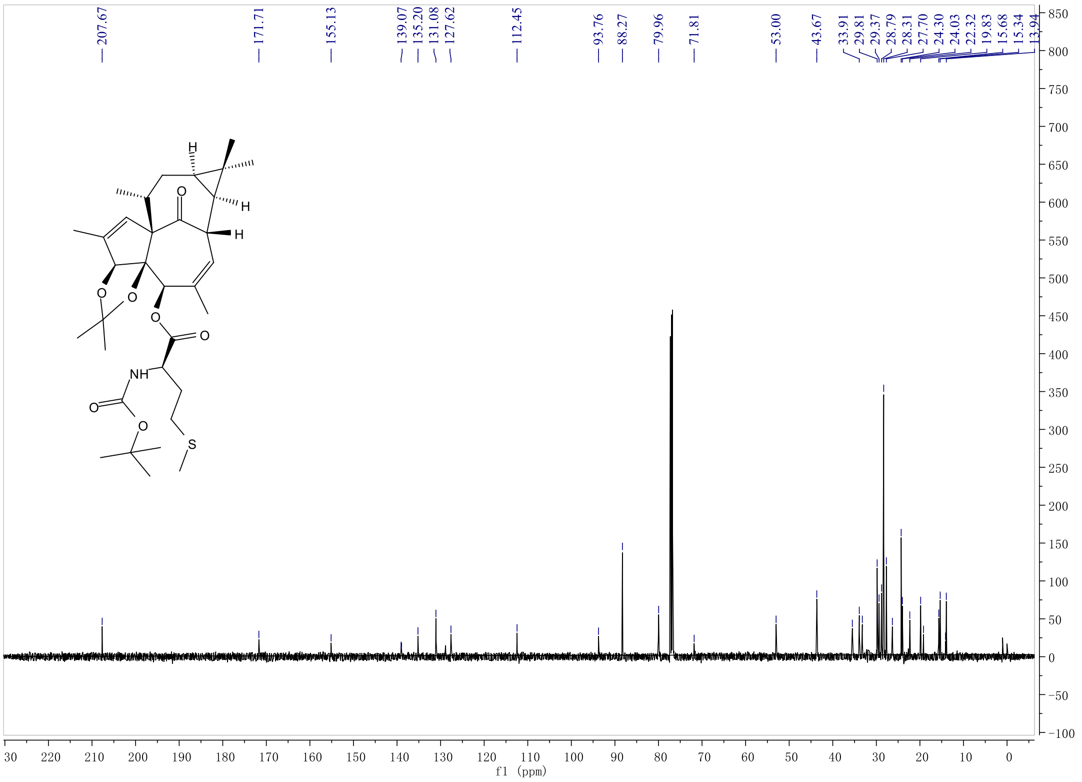

Figure S32. ^{13}C NMR spectra ($CDCl_3$, 125 MHz) of **16**

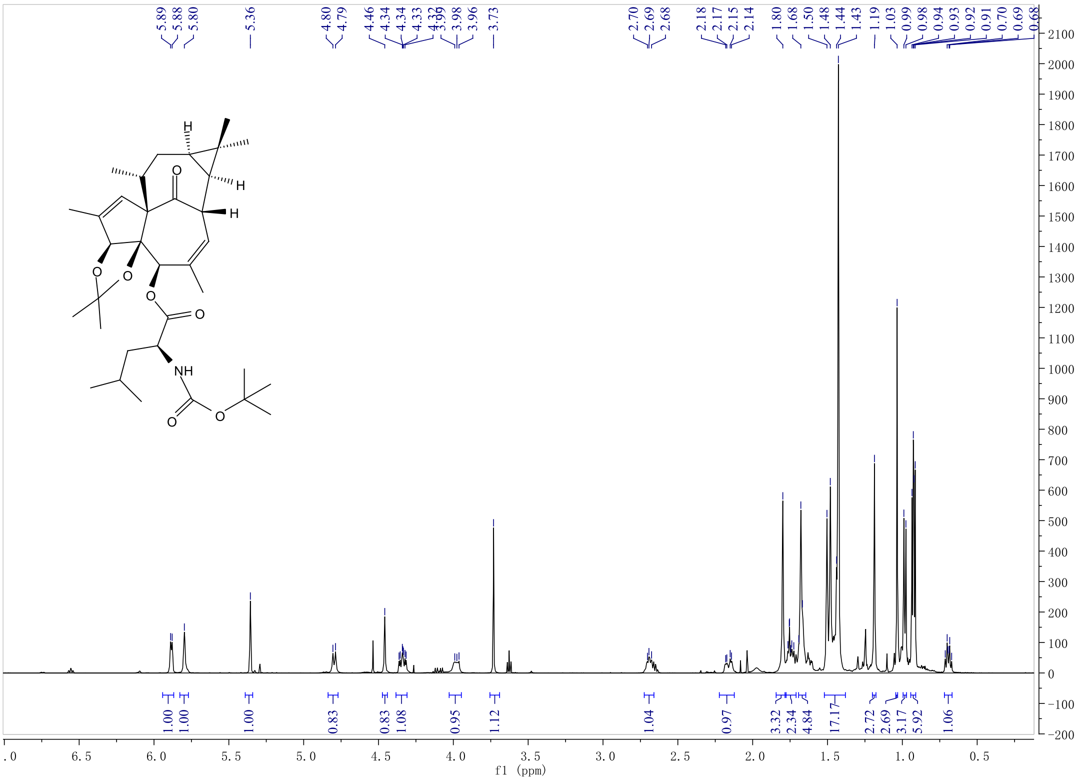

Figure S33. ^{1}H NMR spectra (CDCl$_3$, 500 MHz) of **17**

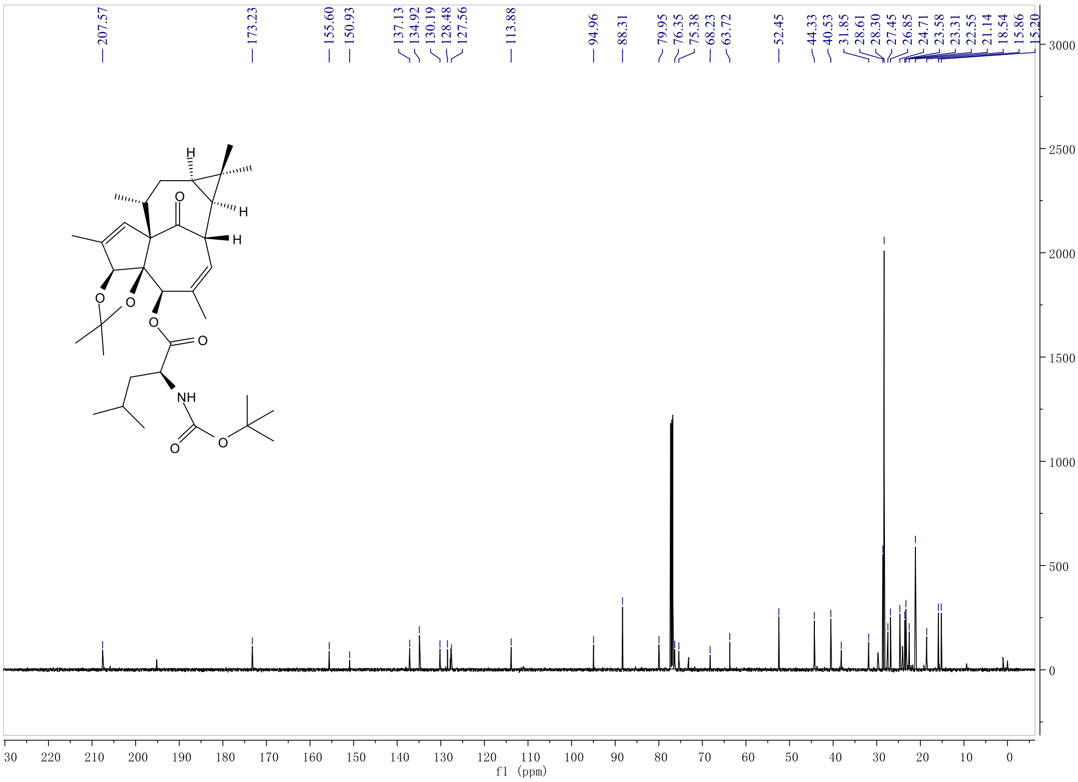

Figure S34. ^{13}C NMR spectra ($CDCl_3$, 125 MHz) of **17**

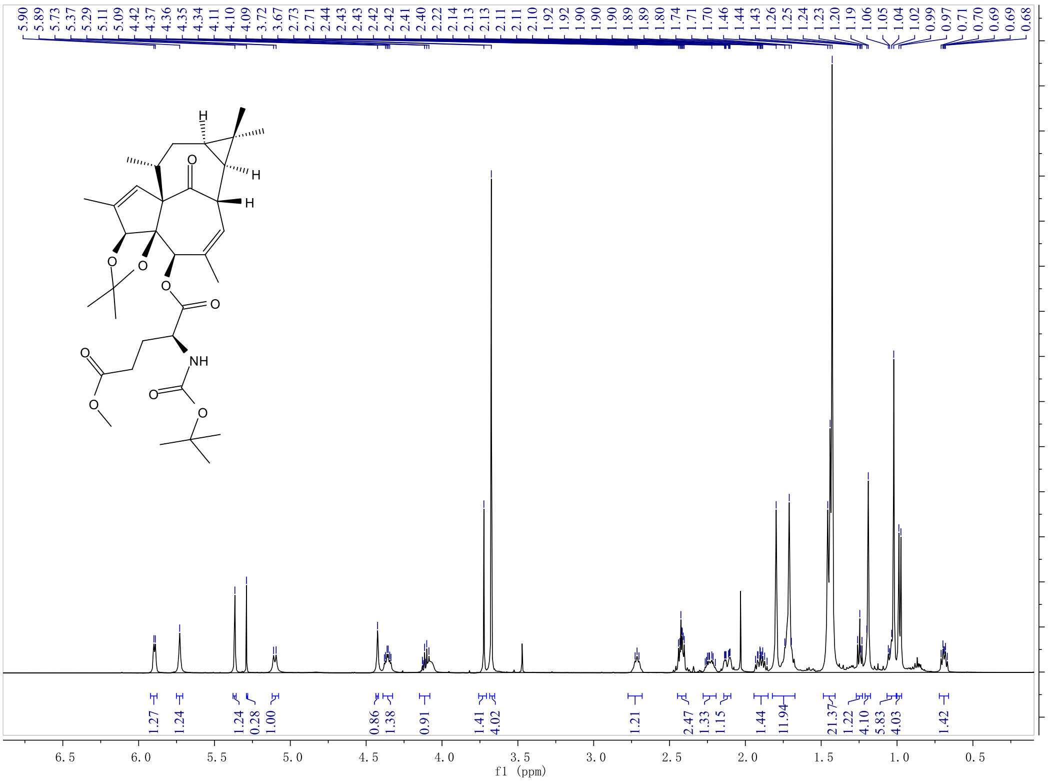

Figure S35. ^{1}H NMR spectra ($CDCl_3$, 500 MHz) of **18**

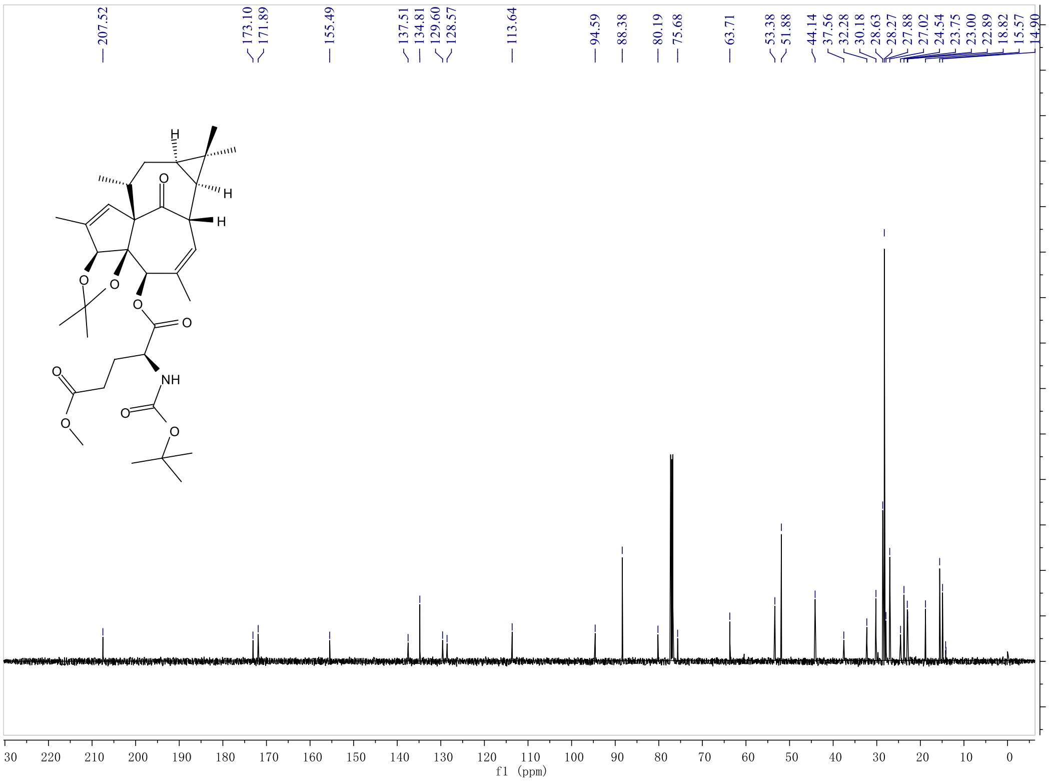

Figure S36. ^{13}C NMR spectra (CDCl$_3$, 125 MHz) of **18**

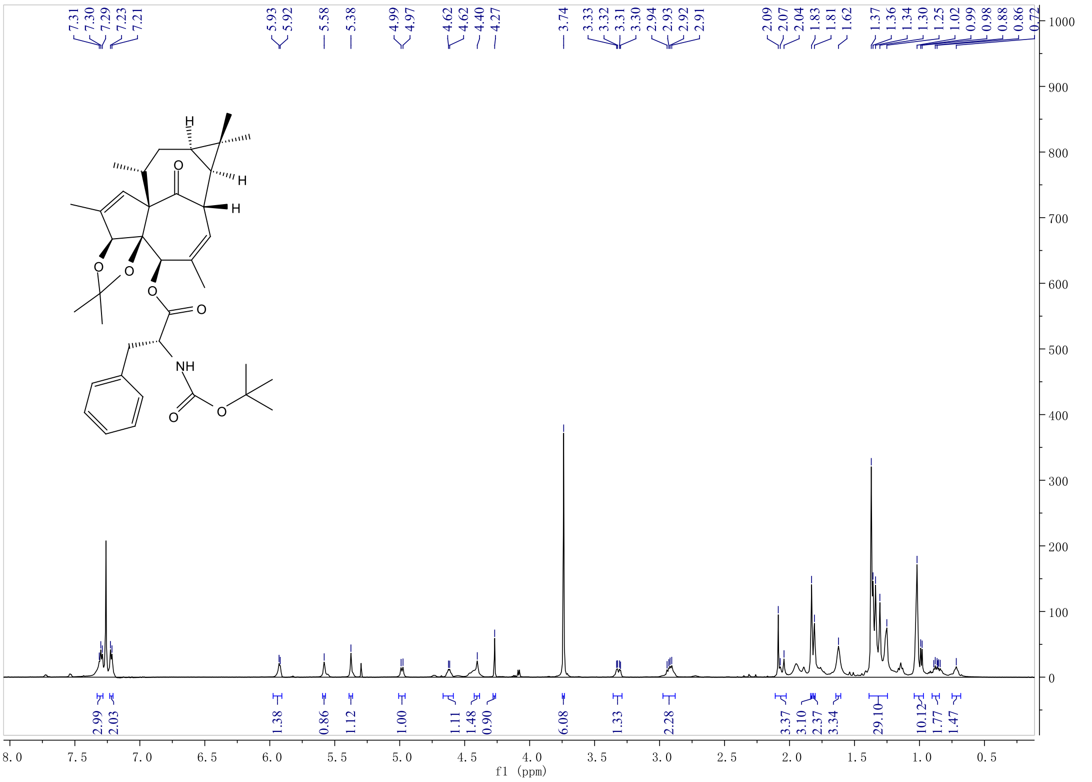

Figure S37. ^{1}H NMR spectra (CDCl$_3$, 500 MHz) of **19**

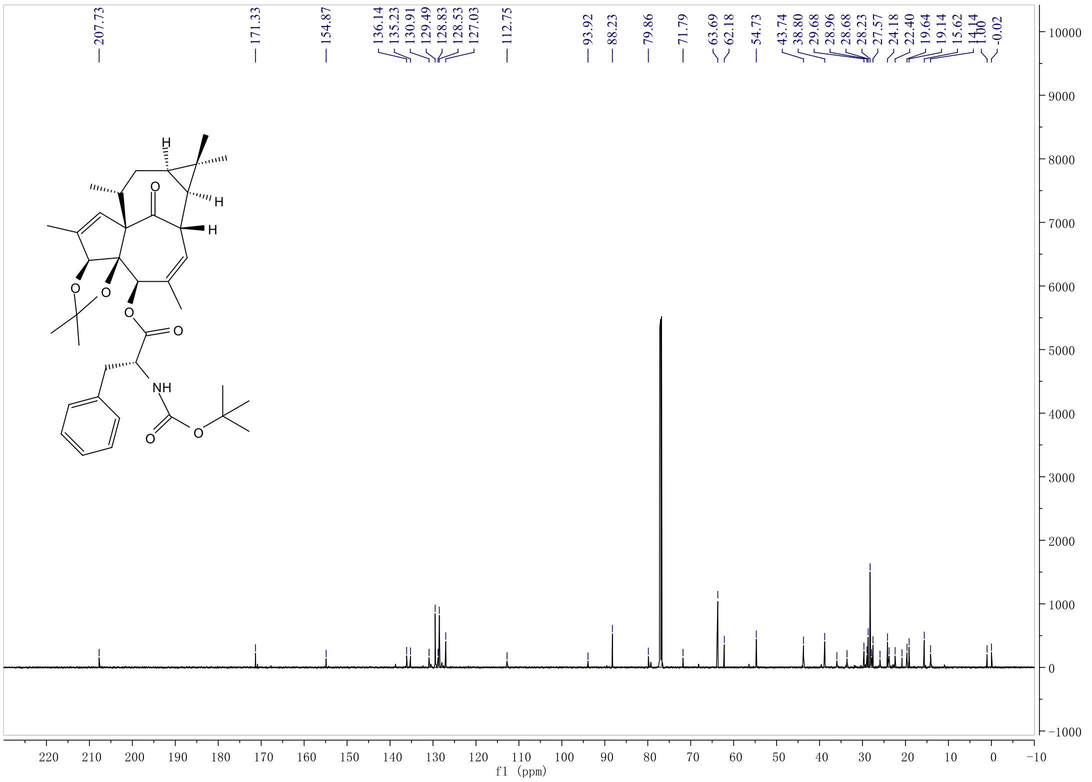

Figure S38. ^{13}**C NMR spectra (CDCl$_3$, 125 MHz) of 19**
